# Interspecies Brain PBPK Modeling Platform to Predict Passive Transport through the Blood–Brain Barrier and Assess Target Site Disposition

**DOI:** 10.3390/pharmaceutics16020226

**Published:** 2024-02-04

**Authors:** Parsshava Mehta, Amira Soliman, Leyanis Rodriguez-Vera, Stephan Schmidt, Paula Muniz, Monica Rodriguez, Marta Forcadell, Emili Gonzalez-Perez, Valvanera Vozmediano

**Affiliations:** 1 Center for Pharmacometrics and Systems Pharmacology, Department of Pharmaceutics, College of Pharmacy, University of Florida, Orlando, FL 32827, USA; parsshava.mehta@ufl.edu (P.M.); amira.soliman@ufl.edu (A.S.); sschmidt@cop.ufl.edu (S.S.); 2 Department of Pharmacy Practice, Faculty of Pharmacy, Helwan University, Helwan 11795, Egypt; 3 Model Informed Development, CTI Laboratories, Covington, KY 41011, USA; lrodriguez@ctifacts.com (L.R.-V.); pmuniz@ctifacts.com (P.M.); mrodriguez@ctifacts.com (M.R.); 4 Neuraxpharm Pharmaceuticals SL, Clinical Research and Evidence-Generation Science, 08970 Barcelona, Spain; forcadellmarta@gmail.com (M.F.); emiligonzalezperez@gmail.com (E.G.-P.)

**Keywords:** PBPK qualification, BBB permeability, CNS, brain modeling, target site disposition, translational modeling

## Abstract

The high failure rate of central nervous system (CNS) drugs is partly associated with an insufficient understanding of target site exposure. Blood–brain barrier (BBB) permeability evaluation tools are needed to explore drugs’ ability to access the CNS. An outstanding aspect of physiologically based pharmacokinetic (PBPK) models is the integration of knowledge on drug-specific and system-specific characteristics, allowing the identification of the relevant factors involved in target site distribution. We aimed to qualify a PBPK platform model to be used as a tool to predict CNS concentrations when significant transporter activity is absent and human data are sparse or unavailable. Data from the literature on the plasma and CNS of rats and humans regarding acetaminophen, oxycodone, lacosamide, ibuprofen, and levetiracetam were collected. Human BBB permeability values were extrapolated from rats using inter-species differences in BBB surface area. The percentage of predicted AUC and Cmax within the 1.25-fold criterion was 85% and 100% for rats and humans, respectively, with an overall GMFE of <1.25 in all cases. This work demonstrated the successful application of the PBPK platform for predicting human CNS concentrations of drugs passively crossing the BBB. Future applications include the selection of promising CNS drug candidates and the evaluation of new posologies for existing drugs.

## 1. Introduction

The high failure rates involved in developing central nervous system (CNS) drug programs call for an urgent need to review the main reason for treatment failure [1,2,3,4]. The challenges encountered in gaining a quantitative understanding of CNS target site concentrations strongly contribute to this failure [5]. The restrictive nature of the BBB makes it difficult to deliver drugs into the CNS [6]. Passive diffusion is one of the transport mechanisms across the BBB that involves the transfer of drugs and endogenous molecules from the blood to the brain on a concentration gradient. The blood–CSF (BCSF) barrier, formed by the choroid plexus, also contributes significantly to the entry of molecules into the CNS [7]. Therefore, accounting for the entry of molecules through both of these routes is crucial. To forecast this permeability across the BBB, researchers have turned to specific in vitro techniques utilizing cell lines. The most common cell culture models employed to assess permeability are Caco-2, derived from colon carcinoma, and MDCK-MDR1, obtained from Madin–Darby canine kidney cells transfected with the human MDR1 gene. These cell lines are utilized to predict brain permeability [8,9,10,11,12], albeit with the limitation of requiring suitable scaling factors, or careful considerations of downregulation of certain properties in vitro which alter BBB permeability when performing in vitro–in vivo extrapolations for accurate predictions. The utilization of these scaling factors is crucial for optimization, primarily due to the nature of Caco-2 cancer cell lines, which are rapidly proliferating cells. This can result in biased estimates, as the integrity of their cell layers may differ from that of the BBB in vivo.

Microdialysis has emerged as a significant technique that allows serial sampling of interstitial fluid from the same animal and across multiple physiological compartments of the brain and cerebrospinal fluid (CSF). It is the method best suited to characterizing the time profile of drug concentrations in the brain [13]. The major advantage of this technique is its ability to concurrently measure the concentration of unbound drugs in the blood and brain of a single animal over a period of time, without net fluid loss. This sets it apart from other approaches, since variables like drug binding to proteins or components of the brain tissue do not interfere with the movement of the drug across the BBB itself. Overall, this time–concentration profile obtained from in vivo microdialysis experiments can provide valuable information.

Physiologically based pharmacokinetic (PBPK) modeling has been successfully used from the early stages of drug development to predict time-dependent drug profiles in various body tissues based on in vitro input data and molecular and physicochemical properties of the drug [14,15]. The increasing number of PBPK modeling submissions over the last decade culminated in draft guidance by both the European Medical Agency (EMA) [16] and the US Food and Drug Administration (FDA) [17]. The importance of PBPK modeling in the realm of predicting the target site concentrations when they are difficult to measure or involve invasive methods is growing traction. For instance, Aulin et al. [18] highlight the significance of the pulmonary PBPK model in characterizing target site exposure to better understand its antimicrobial effects. Similarly, a study by Eigenmann et al. [19], which shows the importance of antibody binding to tumor cells in eliciting their response, used PBPK modeling and further evaluated its impact on target affinity on tumor accumulation. Establishing confidence in PBPK models is crucial, but challenges such as poor in vitro–in vivo correlations, parameter non-identifiability, and lack of validation of predictive performance persist [20].

**Figure 1:**
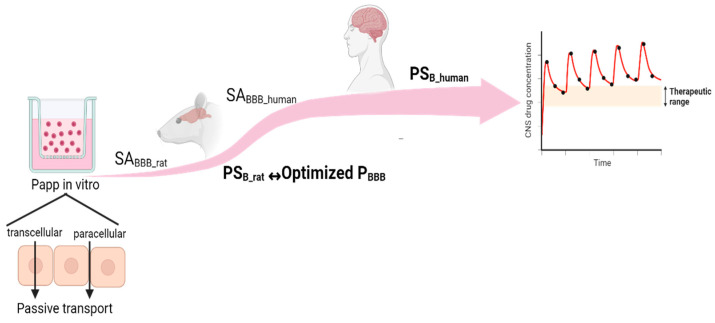
Summary of in vitro–in vivo extrapolation for scaling in vitro-derived permeability to the in vivo BBB of rats and humans. BBB: blood–brain barrier, SA: surface area, PS: permeability surface area product.

The disparities in drug exposures in the brain across species cannot be adequately explained by simple allometric scaling, as it only considers differences in body size and neglects variations in body weight to brain weight ratios. Additionally, as noted by Sharma et al., “the membrane permeability of a drug is a property unaffected by size and stays relatively constant across species” [21]. To address this limitation of allometry, the application of physiologically based pharmacokinetic (PBPK) concepts becomes crucial. PBPK allows for the substitution of physiological parameter values for preclinical species with their corresponding human values, leveraging widely available data in the literature [22]. This increasingly popular approach of using in vitro data within the PBPK model to make predictions of in vivo systems (animals and humans) has been successful, since it provides a more mechanistic basis for interspecies translation of preclinical models [22]. For example, Kielbasa et al. demonstrated the interspecies translation of BBB penetration to provide a prediction of unbound brain concentrations in humans using a model consisting of separate compartments for blood, brain extracellular fluid (ECF), brain intracellular space, and cerebrospinal fluid (CSF) [23]. As emphasized by Ball et al. (2013), “It can be considered that passive diffusion across biological membranes is similar between species when differences in their respective surface areas are taken into account” [8]. This indicates that BBB permeability can be considered as a drug-specific parameter and thus scaled from rats to humans. Hence, permeability measured across in vitro monolayers for passively transported drugs or in the presence of suitable inhibitors of active transport for transporter substrates can be used in human PBPK models, as schematized in Figure 1.

Recognizing the limitations inherent to in vitro parameters and the need to overcome challenges tied to the use of empirical scaling factors, we developed and qualified a robust model platform tailored for compounds that traverse the BBB devoid of active transporters’ involvement in humans. The proposed methodology employs the use of experimental data from neuropharmacokinetic experiments to optimize the BBB permeability value using the rat PBPK model. Subsequently, this optimized value is then scaled to humans, accounting for differences in surface area between the species to make reliable and more accurate predictions in the CNS. The work presented here aimed to qualify the platform model to be used as a tool to predict passive CNS transport when ECF/CSF concentrations are sparse or unavailable in humans. To substantiate the model’s performance, qualification has been conducted using five drugs—acetaminophen, oxycodone, lacosamide, ibuprofen, and levetiracetam—for which data for both rats and humans are available in the literature. The drugs are specifically selected with logP values ranging from −0.67 to 3.97. This range covers a wide spectrum, encompassing both hydrophilic and hydrophobic compounds capable of crossing the BBB. Thus, acknowledging the importance of validation of these predictive tools for untested scenarios and building confidence in prospective predictions is vital. This approach represents a promising stride towards a better understanding of target site disposition, supporting more accurate drug development within the challenging realm of CNS-targeted therapeutics.

## 2. Materials and Methods

**Figure 2:**
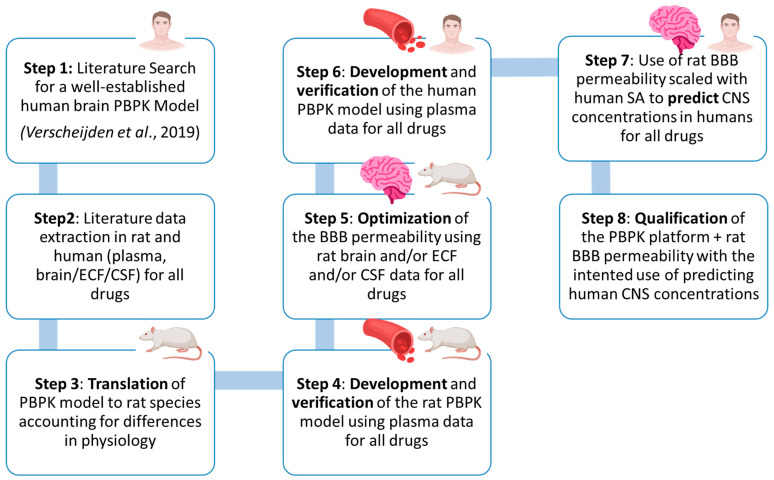
Model framework development and verification workflow of acetaminophen, oxycodone, lacosamide, ibuprofen, and levetiracetam. PBPK: physiologically based pharmacokinetic model, ECF: extracellular fluid, CSF: cerebrospinal fluid, BBB: blood–brain barrier, SA: surface area, CNS: central nervous system [28].

A comprehensive overview of the model platform’s qualification with acetaminophen, oxycodone, lacosamide, ibuprofen, and levetiracetam is provided in Figure 2. A detailed description of each component of the workflow is mentioned in the sections below.

### 2.1. Software

Different aspects of the study were facilitated using the following software:

- PBPK Model Development: Pumas^®^ version 2.2.0 [24] (PumasAI, Dover, DE, USA) an integral package within the Julia programming language, is used to develop the PBPK models for both rat and human subjects.
- PK Metrics’ Calculation: The NCA package within the Pumas^®^ environment was used for computation of pharmacokinetic (PK) metrics.
- Data Management and Visualization: To effectively manage and visualize our data, we employed R^®^ version 4.2.2 [25], operated through the user-friendly RStudio v2023.06.1 [26].
- Data Extraction from the Literature: Since the data were sourced from existing studies, we employed WebPlotDigitizer [27] (version 4.5) to transform images into plots. This facilitated the extraction of concentration versus time data from said plots for further analysis.

### 2.2. Literature Search and Data Collection

The initial literature search for the best brain PBPK model was conducted using the PubMed search engine. The following search terms (and a combination of them) were used: “PBPK”, “physiologically based pharmacokinetic model”, “brain concentrations”, “neuropharmacokinetics”, “brain pharmacokinetics”, “CSF concentrations” and “CNS delivery” (within the abstract or title of the manuscript). After a thorough search, a brain PBPK model developed by Verscheijden et al., 2019 [28] was chosen as the final model for the proposed methodology and qualification of the platform.

The data collection for the drugs intended for model qualification was based on the following set of criteria:

- Drugs for which passive transport is demonstrated;
- Drugs for which the literature contains rat neuropharmacokinetics studies (published data on plasma and either CSF, ECF, or brain concentrations);
- Drugs for which human plasma, CSF and/or ECF concentrations are available to qualify the brain PBPK model platform.

Taking all of the above criteria into consideration, the following drugs were selected: acetaminophen [29,30], oxycodone [31], lacosamide [31,32,33], ibuprofen [31], and levetiracetam [34]. A summary of studies from the literature in both rats and humans, from which pharmacokinetic data were extracted, is provided in Table 1. The drugs acetaminophen [35], oxycodone [36], lacosamide [37], ibuprofen [38] and levetiracetam [39] have been shown to exhibit linear pharmacokinetics at therapeutic doses, for which simulations were performed. Furthermore, it is noteworthy that the drugs in question encompass a diverse array of Biopharmaceutics Classification Systems (BCSs) as mentioned in Table S3. This characteristic serves to augment the model’s applicability in a more comprehensive manner.

### 2.3. Structure of PBPK Model in Rats and Humans

#### 2.3.1. Whole Body PBPK Model

**Figure 3:**
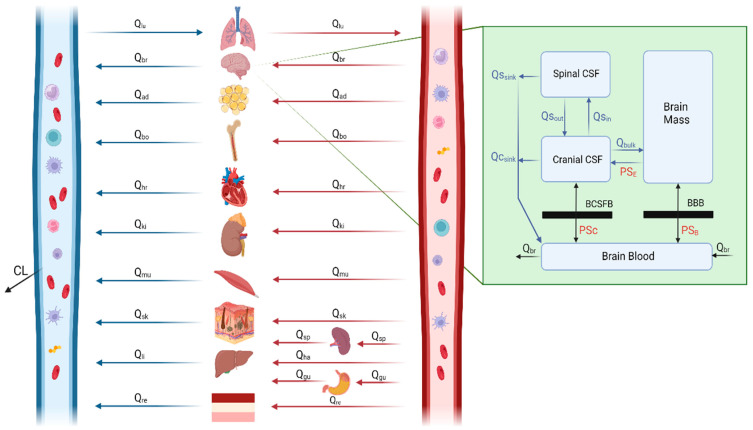
Schematic outline of the brain PBPK platform model, with four brain compartments. Qs_in_ and Qs_out_ represent CSF shuttle flow between cranial CSF and spinal CSF compartments. Qs_sink_ and Qc_sink_ are the flows from CSF compartments to blood. Qbulk represents bulk flow from brain mass to cranial CSF. PS_B_, PS_C_ and PS_E_ represent permeability surface area products between brain blood and brain mass, brain blood and cranial CSF, and brain mass and cranial CSF, respectively. Subscripts lu, br, ad, bo, hr ki, mu, sk, li, re, gu, sp, and ha denote lung, brain, adipose tissue, bone, heart, kidney, muscle, skin, liver, rest tissue, gut, spleen, and hepatic artery, respectively. CL is the total clearance from the model. BBB: blood–brain barrier, BCSFB: blood cerebrospinal fluid barrier.

The whole PBPK model structure, as derived from Vercheijden et al.’s work [28], comprises 14 compartments, encompassing major organs and tissues. Model parameters, including organ volumes and blood flow rates, were obtained from the previously published model and SimCYP [49,50,51]. Subsequently, the model was adapted for rats, accounting for species-specific differences. The equations governing the rat PBPK model were sourced from SimCYP Animal Simulator (version 21 Release 1) [52]. Additionally, the equations for calculating the Kp values using methods such as those of Poulin and Theil (PT), Berezhkovskiy (BZ), and Rodgers and Rowland (RR) were extracted from the relevant literature [53,54,55,56,57] and implemented in the platform model. The model’s structure is visually represented in Figure 3. The various equations for the calculations of the parameters are provided in the Supplementary File under Table S1 for rats and Table S2 for humans.

Drug elimination was incorporated in the model as plasma clearance (CL), using CL values obtained from the literature. Since the goal of the model qualification was mainly to assess the permeability value obtained from in vivo data and predict the human brain concentrations; systemic CL was included as total body clearance and not related to any organ-specific clearance. For drugs given orally, the drug was administered in the gut compartment with a first-order rate of absorption. Alternatively, for drugs administered via the IV route, the administration was simulated in the venous blood compartment.

#### 2.3.2. Brain Model

The 4-compartment permeability limited model (4Brain) is nested within the whole body PBPK model. The 4Brain model consists of the brain blood, brain mass, cranial and spinal CSF compartments. The 4Brain model highlighted in green is shown Figure 3, which represents the various brain compartments. The blood flows within the various compartments are represented as Q_bulk_ (bulk flow from brain mass to the cranial CSF), Qs_in_ and Qs_out_ (representing the CSF shuttle flow between cranial CSF and spinal CSF compartments), Qs_sink_ and Qc_sink_ (flows from the CSF compartment to brain blood). The BBB permeability was optimized for each of the drugs separately and is modelled as PS_B_, as calculated in Equation (1).

(1) $${PS}_{B}=BBB\;Permeability\;(P_{BBB})\;\times \;BBB\;Surface\;Area$$

where ${PS}_{B}$ is the BBB permeability surface area product and is expressed as cm^3^/s (converted to L/min or L/h). The BBB permeability ($P_{BBB}$) is expressed as cm/s and the BBB surface area as cm^2^. The $P_{BBB}$ is a drug-specific parameter that was optimized in rats and then incorporated into the human 4Brain PBPK model, accounting for the difference in BBB surface area between species. The system-specific parameters for the brain sub-compartment are provided in the Supplementary File under Table S1 for rats and Table S2 for humans.

The following model assumptions were made:

- The BBB separates the brain mass from the blood, whereas the BCSFB separates the cranial CSF from blood.
- The compartments are well stirred, with uniform distribution throughout.
- Because the surface area of the BCSFB is 1/10 times that of the BBB [58], the permeability surface area products of BCSFB are one-tenth of the comparable BBB values.
- Because all the model drugs (acetaminophen, oxycodone, lacosamide, ibuprofen and levetiracetam) are not considered substrates for multi-drug transport [28,29,30,31,34] in the BBB or BCSFB, transporter-mediated transfer across these barriers is considered insignificant and hence not incorporated.
- The distribution of the drug in brain mass is assumed to be homogeneous; thus, the ECF concentrations are approximated based on the volume of ECF [30].

### 2.4. Development of the Rat and Human PBPK Model in Plasma

Acetaminophen-, oxycodone-, lacosamide-, ibuprofen-, and levetiracetam-specific drug parameters for the rat and human PBPK models are all outlined in Table S3. Additionally, the Supplementary File included comprehensive information on system parameters within the rat and human PBPK model, accompanied by a sample code for reference. The methodology employed for determining the Kp values was chosen, guided by the following criteria: (1) leveraging previously established PBPK models for analogous drugs; (2) considering physico-chemical properties such as compound type, logP, and pKa; and (3) evaluating the method’s ability to accurately replicate the observed plasma concentrations. The selection of the RR method for acetaminophen and lacosamide was based on the existing PBPK models [31,59]. Similarly, for levetiracetam, the RR method was used based on a previous literature PBPK model of brivaracetam and model fitting [60]. In the case of ibuprofen, the BZ method was opted for, drawing from experiments conducted by the author [61] and the capacity of the method to reproduce the data. As for oxycodone, the PT method was chosen, aligning with the physicochemical properties and the method’s capacity to accurately reproduce the observed data. Drug elimination was incorporated in the model as plasma clearance (CL) with CL values obtained from the literature. CL was 22.8, 49.2, 2.13, 3.88 and 3.96 L/h for acetaminophen, oxycodone, lacosamide, ibuprofen, and levetiracetam, respectively, as provided in Table S3. The inter-individual variability used on the CL and/or Ka (absorption rate constant) parameter was based on existing studies. The inter-individual variability (CV) used on the CL was 35.4, 30, and 42% for acetaminophen, ibuprofen, and levetiracetam, respectively, as provided in Table S3. Uncorrelated variability in CL is included, since total body clearance was not related to any organ-specific or patient characteristics. Thus, it was included in the model as shown below:

Param_indv_ = Param_pop_ × η

where Param_indv_ is the individual parameter’s estimate, Param_pop_ is the average typical value of the population, and η is the variance sampled from a normal distribution.

### 2.5. Optimization of the Brain Permeability from Rat Neuropharmacokinetics Data

The initial value for the optimization of the BBB permeability was obtained from experimental data performed on Caco-2 cells. However, it is crucial to note that Caco-2 cells, originating from human colon carcinoma, may not accurately represent the human blood–brain barrier (BBB). Due to fundamental biological differences, the correlation between Caco-2 cell permeability and in vivo BBB permeability is found to be very low [62]. Consequently, the permeability data from Caco-2 cells were solely utilized as the initial input for the system. Using this input into the PBPK model and brain and CSF or ECF data from the neuropharmacokinetics studies in rats (Table 1), optimization of the BBB permeability value for all five drugs (acetaminophen, oxycodone, lacosamide, ibuprofen, and levetiracetam) was performed using a naïve pooled analysis approach. The naïve pooled analysis treats all observations as coming from a single individual and ignores the inter-individual variations. Thus, this optimization method can be applied to single individuals. No approximations are necessary, since there is no population distribution and hence no joint likelihoods to integrate.

### 2.6. Verification of PBPK Model

The verification of the PBPK models used to predict plasma concentrations in rats and humans and CNS concentrations in rats involved both graphical and numerical assessments. Predicted plasma and CNS concentration profiles were graphed alongside their respective observed data points to allow for visual comparison. Goodness-of-fit (GoF) plots were generated to compare the calculated area under the curve (AUC) and maximum concentration (Cmax) values for both observed and predicted data in rats (plasma and CNS) and humans (plasma). Additionally, for the quantitative evaluation of each independent model for acetaminophen, oxycodone, lacosamide, ibuprofen, and levetiracetam in rats and humans, the geometric mean fold error (GMFE) [63] will be calculated using the following Equations (2) and (3).

(2) $$GMFE=10^{x}\;with\;x=\frac{1}{n}\sum_{i=1}^{n}\left|log10\left(\frac{{A\hat{U}C}_{i}}{{AUC}_{i}}\right)\right|$$

(3) $$GMFE=10^{x}\;with\;x=\frac{1}{n}\sum_{i=1}^{n}\left|log10\left(\frac{{\hat{C}max}_{i}}{{Cmax}_{i}}\right)\right|$$

Here, ${AUC}_{i}$ is the ith observed ${AUC}_{last}$ value, ${A\hat{U}C}_{i}$ is the predicted ${AUC}_{last}$ value and n equals the number of studies. Similarly, ${Cmax}_{i}$ is the ith observed Cmax value, ${\hat{C}max}_{i}$ is the predicted Cmax value, and n equals the number of studies. A GMFE value of 1.25 for predicting Cmax and AUC values was considered a criterion for good model performance in predicting the CNS concentrations.

### 2.7. Qualification of the PBPK Model Platform along with the Optimized BBB Permeability in Rats for Use in Human CNS Predictions

The optimized value of the BBB permeability was then integrated as an input parameter (PS_B_, as shown in Equation (1)) into the human 4Brain PBPK model, accounting for the difference in surface area of BBB, facilitating the prediction of CSF concentrations in humans.

We established a demanding benchmark for success, defining a narrow margin of 1.25-fold, in contrast to the more common 2-fold criteria utilized in PBPK modeling. This stringent criterion aimed to ensure the PBPK model delivers highly reliable predictions of CNS concentrations, thereby reinforcing the robustness and accuracy of our platform. In humans, since CSF is the only accessible fluid for the majority of clinical studies, when the model successfully predicts human CSF concentrations, the predictions of the concentrations in other brain compartments using the model are considered accurate. This is a reasonable consideration based on the scientific literature in the area of PBPK modeling in the CNS [28,30,64].

## 3. Results

### 3.1. PBPK Model Development and Evaluation in Rats

**Figure 4:**
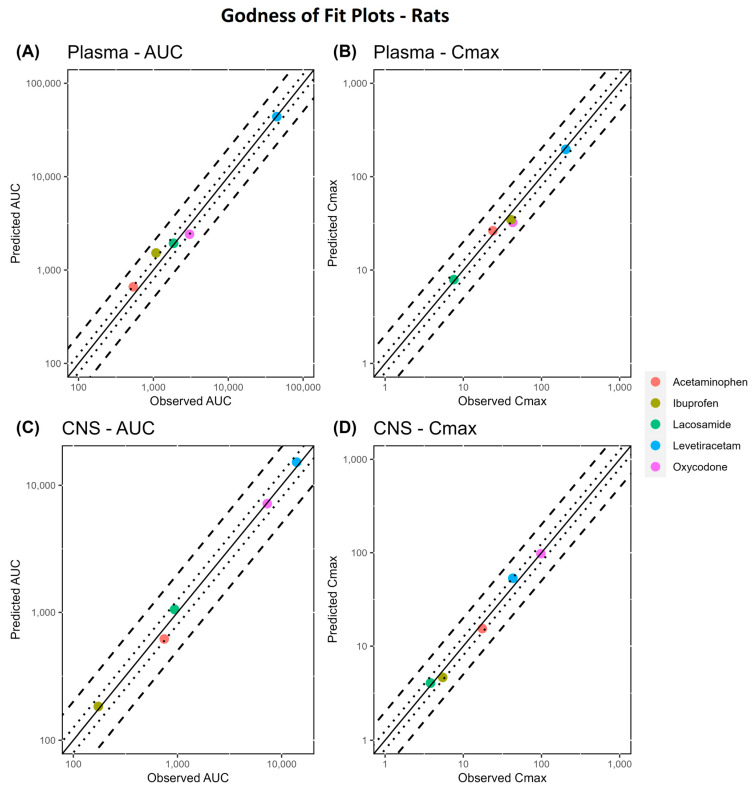
Goodness-of-fit (GoF) plots of predicted versus observed pharmacokinetic metrics (AUC and Cmax) in the plasma (panel (**A**,**B**)) and CNS (panel (**C**,**D**)) of rats. The line of identity is shown as a solid line; 1.25-fold deviation is shown as a dotted line; and 2-fold deviation is shown as a dashed line.

The whole-body rat PBPK model of acetaminophen, oxycodone, lacosamide, ibuprofen, and levetiracetam was built and comprehensively evaluated using plasma profiles. All model parameters are provided in Tables S1 and S3. The optimized P_BBB_ value is provided in Table 3. An overlay of the simulated versus observed profiles of both plasma and CNS concentrations is shown in Figure S1. The GOF plots illustrate the alignment between the predicted versus observed pharmacokinetic metrics, specifically AUC and Cmax, as shown in Figure 4. Additionally, for levetiracetam, as ECF concentrations were used after 40 mg/kg to predict the P_BBB_, an external verification was carried out to confirm the validity of the optimized value using rat ECF (80 mg/kg) and CSF (20–80 mg/kg) data (Table 1). The results are depicted in Figures S2 and S3.

The GMFE values for AUClast and Cmax values for all studies in rats are shown in Table 2. Across the comprehensive dataset of 20 samples, our predictions met the stringent 1.25-fold acceptance criteria for AUC and Cmax values in 17 samples (85%). The overall GMFE for plasma stood at 1.18 for AUC and 1.14 for Cmax. Similarly, our model showed a consistent accuracy within the CNS, yielding an overall GMFE of 1.10 for AUC and 1.12 for Cmax. Among the remaining three studies with a slightly higher GMFE, two were notable for oxycodone in plasma, reporting GMFE values of 1.26 for AUC and 1.32 for Cmax. Additionally, a single study on ibuprofen exhibited a higher GMFE of 1.41 in plasma. However, it is important to note that all CNS data adhered to the stringent 1.25-fold criteria, highlighting the model’s robust performance in predicting CNS concentrations.

### 3.2. BBB Permeability Values

The initial values from Caco-2 cells (P_app_) and final optimized values for the BBB permeability (P_BBB_) values are shown in Table 3. The final value that is incorporated into the model is the PS_B_, which accounts for the difference in surface area for the species and is also presented in Table 3. It can be seen that for drugs that show an entry delay (i.e., acetaminophen, ibuprofen and levetiracetam) into the CNS, the optimized permeability is lower than that predicted from in vitro experimental data. Conversely, for drugs (oxycodone and lacosamide) that attain their Tmax, similar to the concentrations in plasma, the optimized BBB permeability is higher than the initial experimental value.

### 3.3. PBPK Model Development and Evaluation in Humans

**Figure 5:**
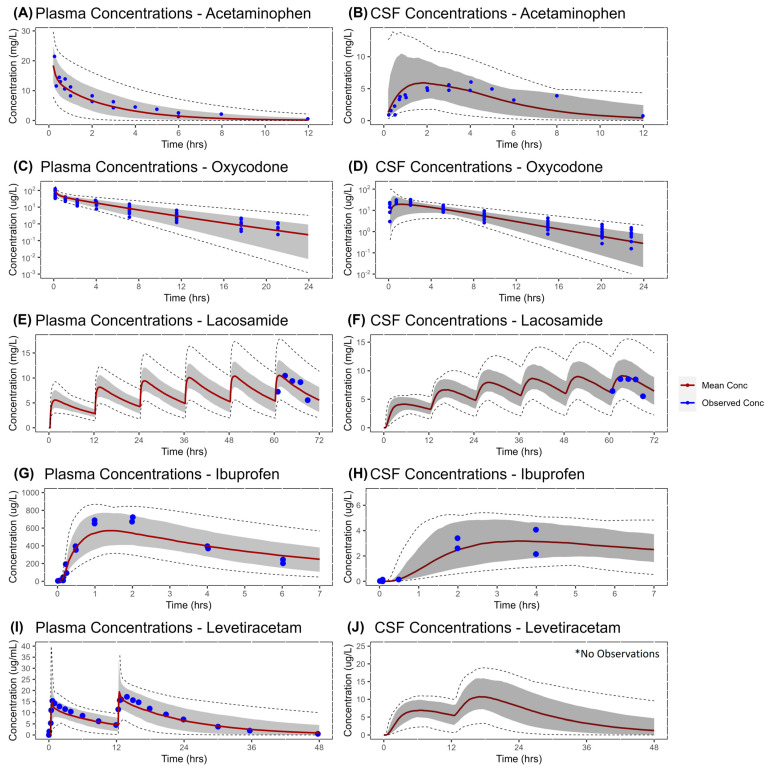
Simulated versus observed concentration profiles for plasma and CNS in humans. Panels (**A**,**C**,**E**,**G**,**I**) represent the plasma concentrations, whereas panels (**B**,**D**,**F**,**H**,**J**) represent the CSF concentrations for acetaminophen, oxycodone, lacosamide, ibuprofen, and levetiracetam, respectively. The red solid line represents the simulated mean concentration profile, the gray shaded area represents the simulated 5th to 95th percentile, and the black dashed lines represent the simulated maximum and minimum. Blue dots represent the observed data from the respective studies: panel (**A**,**B**): Singla et al. [29]; panel (**C**,**D**): Kokki et al. [41]; panel (**E**,**F**): May et al. [43]; panel (**G**,**H**): Brazier et al. [45]; panel (**I**): Rouits et al. [48]. * Only simulations are presented in panel (**J**), as no longitudinal data were available. P–ECF (4.37) and P–CSF (1.14) ratios were used for qualification.

**Figure 6:**
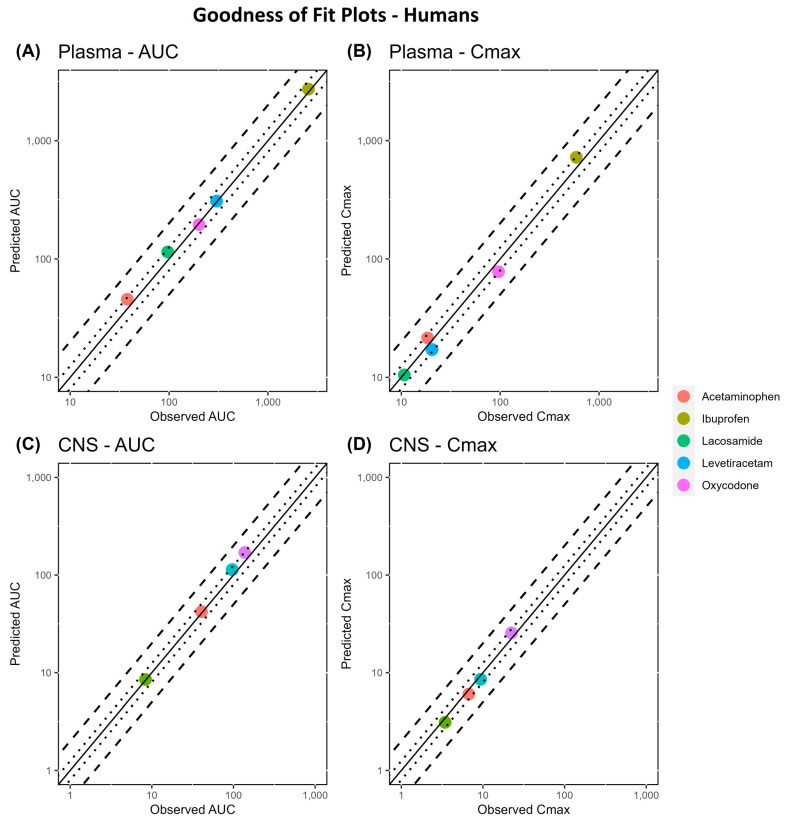
Goodness-of-fit (GoF) plots of predicted versus observed pharmacokinetic metrics (AUC and Cmax) in the plasma (panel (**A**,**B**)) and CNS (panel (**C**,**D**)) of humans. The line of identity is shown as a solid line; 1.25-fold deviation is shown as a dotted line; and 2-fold deviation is shown as a dashed line.

The final PS_B_ value as derived from Equation (1) is shown in Table 3. The system parameters and equations are provided in Table S2, and all drug-specific parameters are provided in Table S3. An overlay of the simulated versus observed plasma profiles of both plasma and CNS concentrations for the drugs acetaminophen, oxycodone, ibuprofen, lacosamide, and levetiracetam are shown in Figure 5. The GOF plots illustrate the alignment between the predicted versus observed pharmacokinetic metrics, specifically AUC and Cmax, as shown in Figure 6. Due to limited information relating to the dosing regimen of levetiracetam [47] and the lack of longitudinal concentration–time data, the verification of CSF concentrations of the model was performed based on the published ratio of plasma–brain extracellular fluid (P:ECF) and plasma–cerebrospinal fluid (P:CSF). Considering that levetiracetam crosses the BBB via passive diffusion, no non-linearity issues are expected due to differences in the drug posology between studies. The mean reported values of P–CSF and P–ECF were 1.14 and 4.37, respectively [47], which were compared with the mean AUC ratios of P–CSF and P–ECF.

The GMFE values for AUClast and Cmax values for all studies in humans are shown in Table 4. Across the comprehensive dataset of 20 samples, our predictions met the stringent 1.25-fold acceptance criterion for AUC and Cmax values in 20 samples (100%). The overall GMFE for plasma was 1.10 for AUC and 1.17 for Cmax. Similarly, our model showed a consistent accuracy within the CNS, yielding an overall GMFE of 1.10 for AUC and 1.11 for Cmax. Notably, all CNS data precisely adhered to the rigorous 1.25-fold criterion, underscoring the robust performance of our model in predicting concentrations within the CNS.

## 4. Discussion

There is a need for qualified brain PBPK models that are able to predict the distribution of drugs within the CNS. This task is non-trivial due to the lack of human data and clear qualification standards. In order to overcome this data challenge, we developed and qualified a brain PBPK model in rats in a stepwise manner; this model was then translated to humans by focusing on five drugs (acetaminophen, oxycodone, lacosamide, ibuprofen and levetiracetam) that passively distribute into the brain [29,30,31,34]. The 4Brain PBPK model was able to describe the data well based on the low values of the GMFEs of AUC and Cmax both in plasma and CSF, which fall below the stringent1.25-fold criterion. In the current methodology, data from rat neuropharmacokinetic studies along with the PBPK model were utilized to optimize the BBB permeability value for the drugs in rats using the in vitro permeability derived from Caco-2 cell lines as the initial input. The predictive capacity of the optimized value of the BBB permeability obtained from rats was then evaluated to predict CNS concentrations in humans. Therefore, the initial hurdle of accurately predicting the brain exposure is overcome because there is no need for an empirical scaling factor to account for the difference between Caco-2 experimental values, since the BBB permeability is obtained from optimizing in vivo neuropharmacokinetic data. When comparing the optimized BBB permeability values to experimental values, it was observed that using Caco-2 cell experimental values in the PBPK model initially led to either overprediction or underprediction of cerebrospinal fluid (CSF) concentrations. Since Caco-2 cells originate from human colon carcinoma and have a high variability [67] associated with them, they may not accurately represent the human blood–brain barrier (BBB). Due to fundamental biological differences, the correlation between Caco-2 cell permeability and in vivo BBB permeability was found to be very low [62]. To help overcome these differences, Fenneteau et al. [68] used a scaling factor of 150 in a whole-body mouse PBPK, as suggested by Pardridge [69] and coworkers for correlation of apparent permeability measured in bovine brain capillary endothelial cells to in vivo data for a dataset of 13 passively permeable drugs. For instance, in Table 3, the ratios of optimized vs. experimental permeability values for drugs like acetaminophen (0.35), ibuprofen (0.66), and levetiracetam (0.06) were lower, indicating a delay in the time taken to reach peak concentrations (Cmax) in the CSF compared to plasma. Conversely, higher ratios were observed for drugs like oxycodone (1.58) and lacosamide (98.12). This disparity may be attributed in part to the static nature of Caco-2 cell experiments. In contrast, BBB cells in in vivo experiments are exposed to shear stress induced by blood flow, which plays a crucial role in regulating barrier function [70]. Overall, since in vitro methods are not better at predicting in vivo BBB permeability [71,72,73,74], the current methodology of predicting the BBB from rat observations is proposed. This dynamic aspect of using in vivo experimental data contributes to a more accurate representation of drug permeability across the BBB and, subsequently, CNS drug concentrations. The goal of this translation approach has practical applications in drug development to study the pharmacokinetics of drugs at the target site of action in the CNS. In the future, aligning with the FDA’s emphasis on replacing, reducing, and refining reliance on animal testing, advancements such as employing microfluidic-based in vitro models that incorporate stem cell-derived endothelial cells, along with primary astrocytes, pericytes, and neurons, hold promising potential for more accurately predicting blood–brain barrier (BBB) permeability [75].

Having a model platform that considers all aspects of BBB permeability can be challenging, and establishing this in one go is unrealistic. So, in this initial step, the current work focuses exclusively on drugs which cross the BBB via passive transport, i.e., those that are devoid of active transporters. The relevance of the BBB permeability value optimized using rat neuropharmacokinetic data is supported by the scientific literature. Permeability-limited drugs will have their transport rate affected by the drug itself (i.e., their permeability, P) and by the surface area of the brain capillaries (S), with both characteristics being combined in a single parameter, PS. The capillary surface area values reported in rats are consistently in the 100–150 cm^2^/g brain range. A more detailed study showed that the capillary surface area varies between brain regions, the highest value being obtained in the cortical grey matter. Human data are broadly similar to rat data, with 100–200 cm^2^/g brain, depending on the region considered. According to Karbowski [76], the fraction of the capillary volume is invariant with respect to brain volume, which would indicate a capillary surface area (expressed per gram or ml brain) conserved across species. The non-P-gp substrate diazepam has a brain PS of 2.6 mL/min/g in human patients [77], which is indistinguishable from the value of 3.0 mL/min/g reported in rats [78]. This finding confirms similar S values (once expressed per gram tissue) between rodents and humans, assuming P is maintained throughout the species. A set of 21 diverse compounds were tested for their brain capillary permeability PS in mice and rats, using in situ brain perfusion [79]. The measured PS values were similar between the two species with only one noticeable exception, vincristine, presumably because of bias due to active transport. In another study, felbamate was found to have a broadly comparable brain PS in mouse, rat, and rabbit (~0.20, 0.09, and 0.17 mL/min/g, respectively). The brain pharmacokinetics of selected opioids has been measured in mouse and compared to clinical data [80]. The values of the brain equilibration half-lives of the non-P-gp substrates alfentanyl, sulfentanyl, and fentanyl were found to be remarkably analogous in mouse and humans. Additionally, a strong interspecies correlation between unbound serum/plasma EC50 suggests that brain distribution characteristics are similar between humans and mice [80,81]. These finding would further confirm that PS values (mL/min/g) are conserved between the two species. Taken collectively, all the data available so far suggest that rodent values of PS should be appropriate for human PBPK modelling for drugs accessing the CNS via passive transport.

A CNS PBPK model initially developed by Westerhout et al. [30] for acetaminophen used a rat model to predict clearance into the brain; this clearance is a product of the permeability value (drug-specific) and surface area (system-specific). Predicting value for both of these parameters combined into one value makes scaling of these parameters to humans difficult, and hence makes predictions in the CNS less reliable. This model was then further refined by Yamamoto et al. [82], and a final revision to this model by Saleh et al. [83] was developed; still, this model accounted for only a unidirectional flow in CSF compartments. This assumption was based on an earlier theory called “third circulation” [84], which states that CSF is only produced by choroid plexus, and thus the movement of drug in the CSF compartments was unidirectional. This assumption was further challenged and corrected in the Lei-CNS 3.1 model to account for the bidirectional CSF flow [85]. Thus, overall, among all the CNS PBPK models discussed in the literature, the model by Vercheijden et al. [28] was chosen because it has been scaled from adults to pediatric patients for acetaminophen, ibuprofen, naproxen, and meropenem over a wide age range, which implies the model structure is robust. Moreover, this model can be employed to incorporate various pathophysiological changes that occur in disease conditions which alter the permeability of the BBB. Nevertheless, the authors used CSF concentrations in humans to optimize the value of BBB permeability. The approach proposed here adds the translational component to this CNS model platform, allowing us to derive concentrations in the CNS site of action when no or very limited CNS concentrations are available in humans for drugs that passively cross the BBB. Therefore, we expanded the application to inform the selection of promising drug candidates using preclinical data as well as assessing different dosing regimens and products for established drugs. The translation of the PBPK model to rats was achieved by accounting for the differences between the species physiology, the code for which is provided in the Supplementary File. The different Kp methods used to predict the distribution of the drug have been also incorporated, allowing the selection of the best model based on the compound type [53,54,55,56,57,86]. However, it is noteworthy that the current model does not encompass drugs that traverse the BBB via transporters, primarily due to limitations in transporter expression across different species. Studies by Hoshi et al. [87] have shown a three-fold higher P-gp expression in the human BBB as compared to the rodent BBB. In another study by Verscheijden et al. [31], the researchers found a difference in Kp_brain_ ratios of ≥2-fold between human and mice species. In our rat PBPK model for lacosamide, a drug efflux parameter at the BBB had to be incorporated to effectively capture the CNS concentrations. On the contrary, this efflux at the BBB was not incorporated for the human CNS PBPK model, wherein only the permeability by passive diffusion was scaled, leading to predictions in the CNS that were well within the range of 1.25-fold, indicating the lack of transporter effect in humans as previously demonstrated and supported by very high oral bioavailability (~100%) [31,32,33].

In humans, CSF drug concentrations are mainly available from pharmacokinetic trials, due to the limitation of accessing other brain compartments. CSF data were thus used to qualify the performance of this translational approach. CSF cannot be considered a relevant compartment for antibiotics, antiepileptic, or analgesic drugs in which the concentration in the parenchymal extracellular fluid is probably more important [85]. Likewise, a concentration gradient exists between CSF and brain tissue, with the magnitude being determined by the relation of CSF bulk flow [88]. This phenomenon is well-documented in an example of quinolone antibiotics [89], which reached much lower concentrations in brain ECF compared to CSF, due to the presence of highly efficient efflux transporters at the BBB. Similarly, azidothymidine readily reached the CSF but showed negligible brain uptake, consistent with brain efflux at the BBB [90]. However, such a difference among the compartments is clearly seen in cases with involvement of transporters, and thus our current model is limited to only passive permeability of drugs across the BBB. In case of passive permeability, the assumption is that the drug is equally distributed in the brain, but the drug concentration in the spinal CSF is driven by the CSF production rate, which is the rate-limiting step. Thus, we must make the PBPK model more robust to validate the CSF concentrations, make fluid accessible in humans in majority of cases, and make model predictions of this concentration in other CNS regions. This is a reasonable consideration based on the scientific literature in the area of PBPK modeling in the CNS [30,64]. In addition, for drugs in which homogeneous distribution in the brain can be considered (such as acetaminophen and levetiracetam), concentrations in the ECF can be approximated using the ECF volume. This assumption is taken from the Westerhout [30] brain model, where the volume of the intracellular and extracellular fluid were fixed to the physiological volumes based on total brain volume. As an example, for levetiracetam in rats, ECF concentrations were used to optimize the BBB permeability. Before using this value of permeability in humans, its validity was assessed using rat CSF concentrations from a different scientific study [46]. Exposure in the rat CSF was predicted within a 1.25-fold range, thus supporting the validity of this assumption for levetiracetam.

## 5. Conclusions

The four-compartment brain PBPK model platform demonstrated precise performance in accurately predicting human CSF concentrations (within 1.25-fold) using the BBB permeability value optimized from neuropharmacokinetic experiments in rats without requiring any further fine-tuning, and it is thus considered qualified for this intended purpose. These results underscore the effectiveness of the brain PBPK model for use within various applications for predicting human CNS concentrations with consistent reliability.

## Figures and Tables

**Table 1:** Summary of neuropharmacokinetics studies in rats and humans.

Drugs	Species	Study ID	Dose	Route	n	Weight (g)	Age (y)	CNS Sample	Extra-Vascular Sample
Acetaminophen	Rat	Sauernheimer et al. [40]	25 mg/kg	IV Bolus	6	300–400	-	CSF	Plasma
	Human	Singla et al. [29]	1000 mg	IV	50	-	19–73	CSF	Plasma
Oxycodone	Rat	Ball et al. [8]	0.3 mg/kg	IV Infusion,1 h	11	250–290	-	Brain	Plasma
	Human	Kokki et al. [41]	0.092 mg/kg	IV	11	-	26–60	CSF	Plasma
Lacosamide	Rat	Koo et al. [42]	10 mg/kg	Oral	4	230–260	-	Brain	Plasma
	Human	May et al. [43]	166 mg/12 h	Oral	21	-	18–65	CSF	Plasma
Ibuprofen	Rat	Talhoni et al. [44]	50 mg/kg	Intraperitoneal	3	200–250	-	Brain	Plasma
	Human	Brazier et al. [45]	10 mg	Oral	26	-	55–75	CSF	Plasma
Levetiracetam	Rat	Tong et al. [34]	40 and 80 mg/kg	Intraperitoneal	6	300–350	-	ECF	Serum
	Rat	Doheny et al. [46]	20, 40 and 80 mg/kg	Intraperitoneal	6	250–350	-	CSF	Serum
	Human	Rambeck et al. [47]	-	Oral	3	-	32–44	ECF CSF	Plasma
	Human	Rouits et al. [48]	500 mg	Oral	24	-	18–55	-	Plasma

n: sample size of study, CNS: central nervous system, IV: intravenous, ECF: extracellular fluid, CSF: cerebrospinal fluid.

**Table 2:** Predicted and observed PK metrics of AUC and Cmax for various drugs in rats.

Drug	Sample	AUC_Pred	AUC_Obs	Cmax_Pred	Cmax_Obs	GMFE_AUC	GMFE_Cmax
Acetaminophen	Plasma	538.35	660.62	23.89	26.35	1.23	1.10
Oxycodone	Blood	3040.96	2418.04	42.84	32.37	1.26	1.32
Lacosamide	Plasma	1860.7	1934.89	7.57	7.9	1.04	1.04
Ibuprofen	Plasma	1083.44	1522.96	40.84	34.52	1.41	1.18
Levetiracetam	Plasma	44,542.7	43,927.1	205.06	196.02	1.01	1.05
					**Maximum**	1.41	1.32
					**Minimum**	1.01	1.04
					**GMFE-Plasma**	1.18	1.14
Acetaminophen	CSF	746.57	617.81	17.52	15.42	1.21	1.14
Oxycodone	BM	7279.78	7163.91	98.23	97.7	1.02	1.01
Lacosamide	BM	937.89	1054.89	3.81	4.05	1.12	1.06
Ibuprofen	BM	173.71	183.09	5.45	4.65	1.05	1.17
Levetiracetam	ECF	13,939.6	15,203.7	43.02	53.26	1.09	1.24
					**Maximum**	1.21	1.24
					**Minimum**	1.02	1.01
					**GMFE-CNS**	1.10	1.12

AUC (μg × min/mL): lacosamide, acetaminophen, ibuprofen, AUC (ng × min/mL): oxycodone and AUC (μmol × min/mL): levetiracetam. Cmax (μg/mL): lacosamide, acetaminophen, ibuprofen, Cmax (ng/mL): oxycodone and Cmax (μmol/L): levetiracetam.

**Table 3:** Optimized BBB permeability values.

Drug	Initial Caco-2 Permeability (P_app_) (cm/s)	Optimized Permeability (P_BBB_) (cm/s)	Ratio (P_BBB_/P_app_)	PS_B_ in Rats (ml/min)	PS_B_ in Humans (L/h)
Acetaminophen	31.6 × 10^−6^ [59]	11.11 × 10^−6^	0.35	0.179	5.98
Oxycodone	16.9 × 10^−6^ [31]	26.79 × 10^−6^	1.59	0.434	14.47
Lacosamide	1.916 × 10^−7^ [31]	18.8 × 10^−6^	98.12	0.305	10.15
Ibuprofen	53 × 10^−6^ [65]	35.13 × 10^−6^	0.66	0.57	18.97
Levetiracetam	22.8 × 10^−6^ [66]	1.52 × 10^−6^	0.07	0.024	0.818

P_app_: apparent permeability, P_BBB_: permeability blood–brain barrier, PS_B_: permeability surface area products between brain blood and brain mass.

**Table 4:** Predicted and observed PK metrics of AUC and Cmax for various drugs in humans.

Drug	Sample	AUC_Pred	AUC_Obs	Cmax_Pred	Cmax_Obs	GMFE_AUC	GMFE_Cmax
Acetaminophen	Plasma	37.81	45.58	18.44	21.46	1.21	1.16
Oxycodone	Plasma	201.14	195.01	96.24	77.92	1.03	1.24
Lacosamide	Plasma	96.99	114.49	10.72	10.47	1.18	1.02
Ibuprofen	Plasma	2568.01	2729.26	589.08	723.26	1.06	1.23
Levetiracetam	Plasma	302.52	308.51	20.44	17.14	1.02	1.19
					**Maximum**	1.21	1.24
					**Minimum**	1.02	1.02
					**GMFE-Plasma**	1.10	1.17
Acetaminophen	CNS	40.59	42.11	6.72	6.04	1.04	1.11
Oxycodone	CNS	137.77	170.05	22.35	25.6	1.23	1.15
Lacosamide	CNS	96.55	113.63	9.37	8.55	1.18	1.10
Ibuprofen	CNS	8.37	8.51	3.44	3.11	1.02	1.11
Levetiracetam *	CNS	P:ECF = 4.14 P:CSF = 1.05	P:ECF = 4.37 P:CSF = 1.14	-	-	1.06 (ECF) 1.09 (CSF)	-
					**Maximum**	1.23	1.15
					**Minimum**	1.02	1.10
					**GMFE-CNS**	1.10	1.11

AUC (mg × h/L): acetaminophen, lacosamide and AUC (μg × h/L): oxycodone, ibuprofen. Cmax (mg/L): acetaminophen, lacosamide and Cmax (μg/L): oxycodone, ibuprofen. * Levetiracetam-observed P–CSF (plasma–cerebrospinal fluid) and P–ECF (plasma–extracellular fluid) ratios vs. predicted AUC P–CSF and P–ECF ratios.

## Data Availability

Data are contained within the article and Supplementary Materials.

## Supplementary Materials

The following supporting information can be downloaded at: https://www.mdpi.com/article/10.3390/pharmaceutics16020226/s1, Figure S1: Simulated versus observed concentration profiles for plasma and CNS in rats; Figure S2: Simulated versus observed concentration profiles in rats; Figure S3: Goodness-of-fit plots of predicted vs observed pharmacokinetic metrics (AUC & Cmax) in plasma and CSF/ECF in rats; Table S1: Physiological parameters-Rat PBPK model; Table S2: Physiological parameters-Human PBPK model; Table S3: Physicochemical and Drug Specific Parameters for Acetaminophen, Oxycodone, Lacosamide, Ibuprofen & Levetiracetam. References [8,28,34,40,42,44,46,52,53,56,57,59,91,92,93,94,95,96,97,98,99,100,101,102,103,104,105,106,107,108,109] are cited in the supplementary materials.
