# Supplementary material for: Interspecies Brain PBPK Modeling Platform to Predict Passive Transport through the Blood–Brain Barrier and Assess Target Site Disposition
Source: Pharmaceutics. 2024 Feb 4;16(2):226. doi: 10.3390/pharmaceutics16020226 (PMC10892872; doi:10.3390/pharmaceutics16020226)
Supplement: Supplementary file 1 [file pharmaceutics-16-00226-s001.zip › Supplementary figures and tables.pdf]

Article

Interspecies Brain PBPK Modeling Platform to Predict Passive Transport through the Blood-Brain Barrier and Assess Target-Site Disposition

Supplementary Materials

Parsshava Mehta [1], Amira Soliman [1,2], Leyanis Rodriguez-Vera [3], Stephan Schmidt [1], Paula Muniz [3], Monica Rodriguez [3], Marta Forcadell [4], Emili Gonzalez-Perez [4] and Valvanera Vozmediano [1,3*]

1 Center for Pharmacometrics and Systems Pharmacology, Department of Pharmaceutics, College of Pharmacy, University of Florida, Orlando, FL 32827, USA; parsshava.mehta@ufl.edu (P.M.); amira.soliman@ufl.edu (A.S.); sschmidt@cop.ufl.edu (S.S.)
2 Department of Pharmacy Practice, Faculty of Pharmacy, Helwan University, Helwan 11795, Egypt
3 Model Informed Development, CTI Laboratories, Covington, KY 41011, USA; lrodriguez@ctifacts.com (L.R.-V.); pmuniz@ctifacts.com (P.M.); mrodriguez@ctifacts.com (M.R.)
4 Neuraxpharm Pharmaceuticals SL, Clinical Research and Evidence-Generation Science, 08970 Barcelona, Spain; forcadellmarta@gmail.com (M.F.); emiligonzalezperez@gmail.com (E.G.-P.)
* Correspondence: valva@ufl.edu or vvozmediano@ctifacts.com

Contents

List of Figures

Figure S1: Simulated versus observed concentration profiles for plasma and CNS in rats ------------------- 3

Figure S2: Simulated versus observed concentration profiles in rats -- 5

Figure S3: Goodness-of-fit plots of predicted vs observed pharmacokinetic metrics (AUC & Cmax) in plasma and CSF/ECF in rats --- 6

List of Tables

Table S1: Physiological parameters - Rat PBPK model -- 7

Table S2: Physiological parameters - Human PBPK model --- 10

Table S3: Physicochemical and Drug Specific Parameters for Acetaminophen, Oxycodone, Lacosamide, Ibuprofen & Levetiracetam --- 14

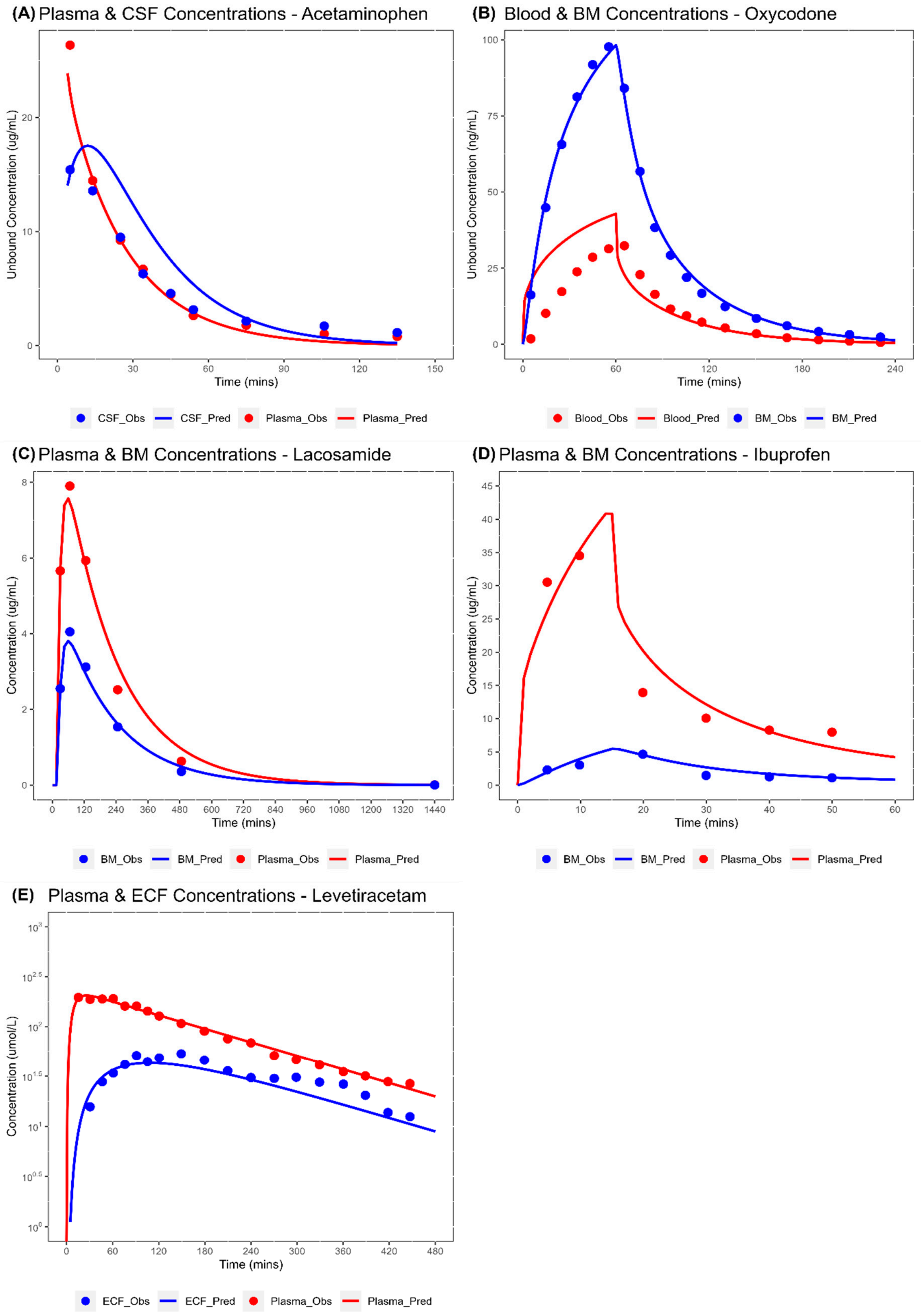
(A) Plasma & CSF Concentrations - Acetaminophen
Unbound Concentration (ug/mL)
Time (mins)
CSF_Obs
CSF_Pred
Plasma_Obs
Plasma_Pred
(B) Blood & BM Concentrations - Oxycodone
Unbound Concentration (ng/mL)
Time (mins)
Blood_Obs
Blood_Pred
BM_Obs
BM_Pred
(C) Plasma & BM Concentrations - Lacosamide
Concentration (ug/mL)
Time (mins)
BM_Obs
BM_Pred
Plasma_Obs
Plasma_Pred
(D) Plasma & BM Concentrations - Ibuprofen
Concentration (ug/mL)
Time (mins)
BM_Obs
BM_Pred
Plasma_Obs
Plasma_Pred
(E) Plasma & ECF Concentrations - Levetiracetam
Concentration (umol/L)
Time (mins)
ECF_Obs
ECF_Pred
Plasma_Obs
Plasma_Pred

Figure S1. Simulated versus observed concentration profiles for plasma and CNS in rats.

Panel A: Red solid line represents the simulated unbound plasma concentrations and blue solid line represents the unbound simulated CSF concentrations for the drug acetaminophen. Red and blue dots represent the observed plasma and CSF concentrations respectively, from Sauernheimer et al [40]

Panel B: Red solid line represents the simulated unbound blood concentrations and blue solid line represents the unbound simulated Brain Mass (BM) concentrations for the drug oxycodone. Red and blue dots represent the observed blood and BM concentrations respectively, from Ball et al [8]

Panel C: Red solid line represents the simulated plasma concentrations and blue solid line represents the simulated BM concentrations for the drug lacosamide. Red and blue dots represent the observed plasma and BM concentrations respectively, from Koo et al [42]

Panel D: Red solid line represents the simulated plasma concentrations and blue solid line represents the simulated BM concentrations for the drug ibuprofen. Red and blue dots represent the observed plasma and BM concentrations respectively, from Talhoni et al [44]

Panel E: Red solid line represents the simulated plasma concentrations and blue solid line represents the simulated ECF concentrations for the drug levetiracetam - 40mg/kg. Red and blue dots represent the observed plasma and ECF concentrations respectively, from Tong et al [34]

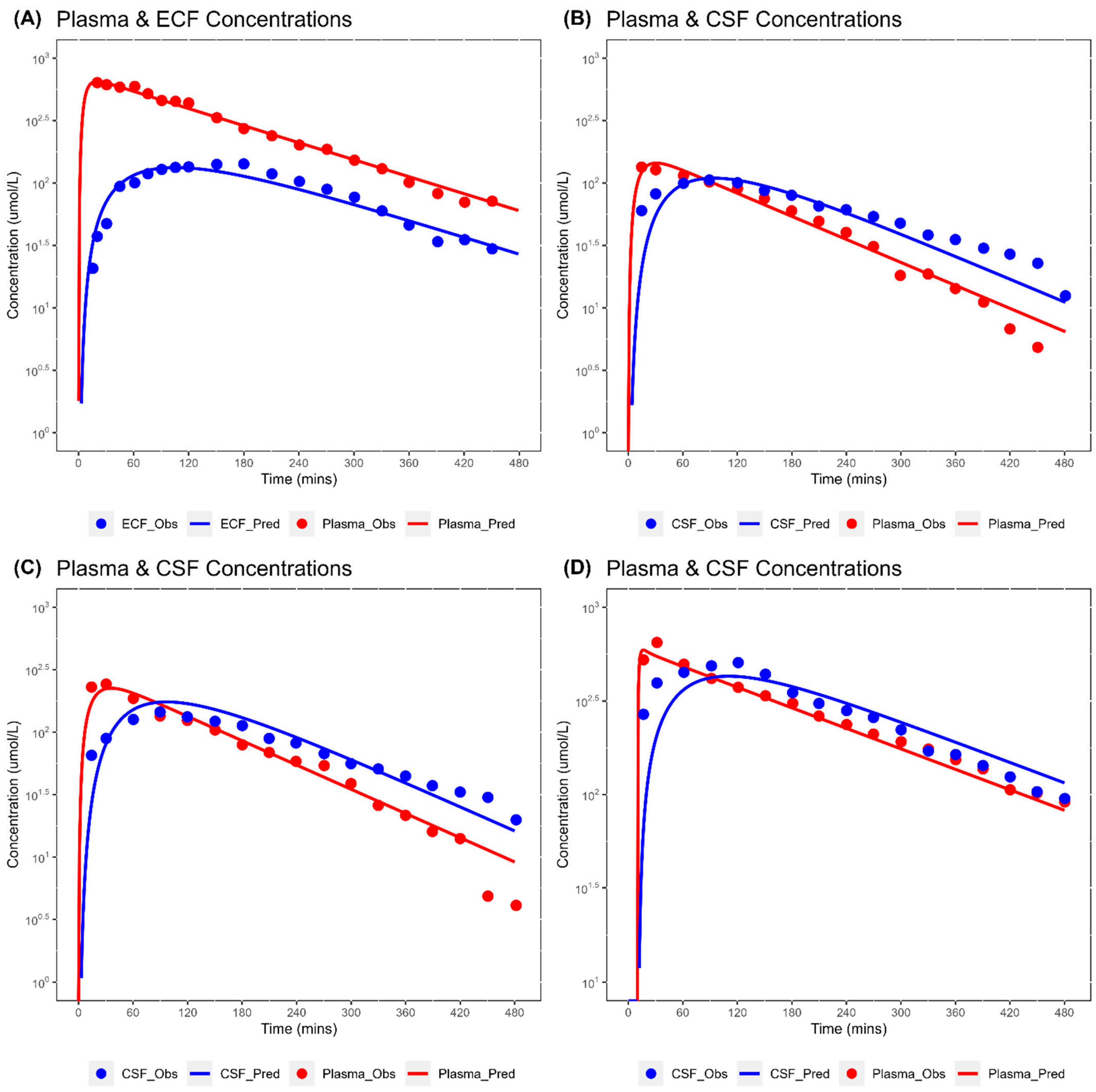

Figure S2. Simulated versus observed concentration profiles in rats. Panel A: Red solid line represents the simulated plasma concentrations and blue solid line represents the simulated ECF concentrations for the drug levetiracetam - 80mg/kg doses from a study by Tong et al. [34]. Red and blue dots represent observed plasma and CSF concentrations, respectively from a study by Tong et al. [34]. Panel B, C & D: Red solid line represents the simulated plasma concentrations and blue solid line represents the simulated CSF concentrations for the drug levetiracetam for the 20mg/kg (B), 40mg/kg (C) and 80mg/kg (D) doses respectively from a study by Doheny et al. [46]. Red and blue dots represent observed plasma and CSF concentrations, respectively for the 20mg/kg (B), 40mg/kg (C) and 80mg/kg (D) doses respectively from a study by Doheny et al. [46].

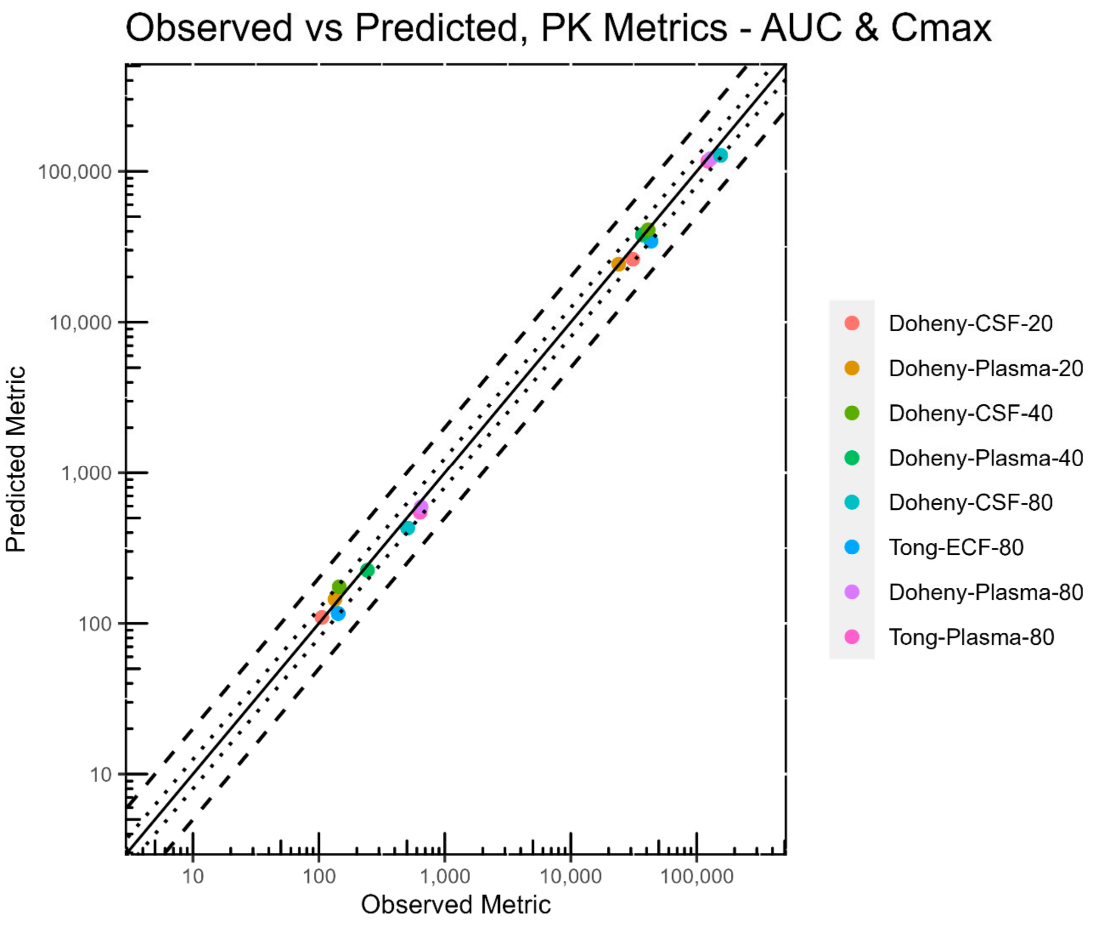

Figure S3. Goodness-of-fit plots of predicted vs observed pharmacokinetic metrics (AUC & Cmax) in plasma and CSF/ECF in rats. The line of identity is shown as a solid line; 1.25-fold deviation is shown as a dotted line; 2-fold deviation is shown as a dashed line. The observed data are from both the above studies Doheny [46], Tong [34].

Table S1. Physiological parameters - Rat PBPK model

Parameter	Equation / Value	Reference
Hematocrit	0.439	[52]
Rat Serum Albumin (g/L)	31.14	[52]
Weight (g)	250	[34]
	**Volumes (mL)**	
**Parameter**	**Equation / Value**	**Reference**
**Other Organ Compartments:**		
Vlung	1.24	[52]
Vadipose	16.66	[52]
Vbone	15.68	[52]
Vheart	1.05	[52]
Vkidney	2.19	[52]
Vmuscle	116.13	[52]
Vskin	39.41	[52]
Vspleen	0.57	[52]
Vgut	6.19	[52]
Vliver	8.57	[52]
Vbla	6.8	[91]
Vblv	13.6	[91]
Vrest	1.3	[91]
**Brain Compartments:**		
Vbrain total	1.24	[52]
Vbrain blood	$0.0310 * Vbtotal$	[52]
Vccsf	$0.144 * Vbtotal$	[52]
Vscsf	$0.18750 * Vccsf$	[52]
Vecf	0.29	[8,52]
Vbrain mass	$Vbtotal - Vccsf - Vscsf$	
	**Fluid flow rates (mL/min)**	
**Parameter**	**Equation / Value**	**Reference**
**Other organ Compartments:**		
Qcarout	80	[52]
Qlung	80	[52]
Qadipose	$5.9 * Qcarout$	[52]
Qbone	$10.1 * Qcarout$	[52]
Qheart	$4 * Qcarout$	[52]
Qkidney	$14.5 * Qcarout$	[52]
Qmuscle	$23.7 * Qcarout$	[52]
Qskin	$5.1 * Qcarout$	[52]
Qspleen	$1.1 * Qcarout$	[52]
Qgut	$10.1 * Qcarout$	[52]
Qha	$1.6 * Qcarout$	[91,52]
Qliver	$Qgut + Qspleen + Qha$	[52]
Qrestflow	1	[52]
**Brain Compartment:**		
Qbrain	1.12	[52]
Qproductionrate	0.0026	[52]

Qbulk	$0.085 * Qproductionrate$			[52]
Qssink	$0.37 * (0.75 * Qproductionrate + Qbulk)$			[52]
Qsout	$0.9 * Qssink$			[52]
Qsin	$(Qssink + Qsout)$			[52]
Qcsink	$(0.75 * Qproductionrate + Qbulk - Qsin + Qsout)$			[52]
**Tissue Composition (fraction)**				
**Parameter**	**Rowdgers and Rowland**	**Poulin and Theil**	**Berezhkovskiy**	**Reference**
Adipose	EW = 0.135<br>IW = 0.017<br>NL = 0.846<br>NP = 0.0016<br>AP = 0.4<br>AR = 0.049<br>LR = 0.068	FW = 0.152<br>NL = 0.846<br>NP = 0.0016<br>pH = 7	FW = 0.152<br>NL = 0.846<br>NP = 0.0016<br>pH = 7	[53,56,57]
Bone	EW = 0.10<br>IW = 0.346<br>NL = 0.017<br>NP = 0.0017<br>AP = 0.67<br>AR = 0.1<br>LR = 0.05	FW = 0.446<br>IW = 0.346<br>NL = 0.017<br>NP = 0.0017<br>pH = 6.7	FW = 0.446<br>IW = 0.346<br>NL = 0.017<br>NP = 0.0017<br>pH = 6.7	[53,56,57]
Gut	EW = 0.282<br>IW = 0.475<br>NL = 0.038<br>NP = 0.0125<br>AP = 2.41<br>AR = 0.158<br>LR = 0.141	FW = 0.757<br>NL = 0.038<br>NP = 0.0125<br>pH = 7.16	FW = 0.757<br>NL = 0.038<br>NP = 0.0125<br>pH = 7.16	[53,56,57]
Heart	EW = 0.32<br>IW = 0.456<br>NL = 0.014<br>NP = 0.0111<br>AP = 2.25<br>AR = 0.157<br>LR = 0.16	FW = 0.776<br>NL = 0.014<br>NP = 0.0111<br>pH = 7.13	FW = 0.776<br>NL = 0.014<br>NP = 0.0111<br>pH = 7.13	[53,56,57]
Kidney	EW = 0.273<br>IW = 0.483<br>NL = 0.012<br>NP = 0.0242<br>AP = 5.03<br>AR = 0.13<br>LR = 0.137	FW = 0.756<br>NL = 0.012<br>NP = 0.0242<br>pH = 7.31	FW = 0.756<br>NL = 0.012<br>NP = 0.0242<br>pH = 7.31	[53,56,57]
Liver	EW = 0.161<br>IW = 0.573<br>NL = 0.014<br>NP = 0.024<br>AP = 4.56<br>AR = 0.086<br>LR = 0.161	FW = 0.734<br>NL = 0.0138<br>NP = 0.024<br>pH = 7.03	FW = 0.734<br>NL = 0.0138<br>NP = 0.024<br>pH = 7.03	[53,56,57]
Lung	EW = 0.336<br>IW = 0.446	FW = 0.782<br>NL = 0.0219	FW = 0.782<br>NL = 0.0219	[53,56,57]

	NL = 0.022<br>NP = 0.0128<br>AP = 3.91<br>AR = 0.212<br>LR = 0.168	NP = 0.0128<br>pH = 7.36	NP = 0.0128<br>pH = 7.36	
Muscle	EW = 0.118<br>IW = 0.63<br>NL = 0.01<br>NP = 0.0072<br>AP = 1.53<br>AR = 0.064<br>LR = 0.059	FW = 0.748<br>NL = 0.01<br>NP = 0.0072<br>pH = 6.27	FW = 0.748<br>NL = 0.01<br>NP = 0.0072<br>pH = 6.27	[53,56,57]
Skin	EW = 0.382<br>IW = 0.291<br>NL = 0.06<br>NP = 0.0044<br>AP = 1.32<br>AR = 0.277<br>LR = 0.096	FW = 0.673<br>NL = 0.06<br>NP = 0.0044<br>pH = 6.27	FW = 0.673<br>NL = 0.06<br>NP = 0.0044<br>pH = 6.27	[53,56,57]
Spleen	EW = 0.207<br>IW = 0.579<br>NL = 0.0077<br>NP = 0.0113<br>AP = 3.18<br>AR = 0.097<br>LR = 0.207	FW = 0.786<br>NL = 0.0077<br>NP = 0.0113<br>pH = 6.27	FW = 0.786<br>NL = 0.0077<br>NP = 0.0113<br>pH = 6.27	[53,56,57]
Plasma	EW = 0.945<br>IW = 0.0<br>NL = 0.0023<br>NP = 0.0013<br>AP = 0.057<br>AR = NA<br>LR = NA	FW = 0.945<br>NL = 0.0023<br>NP = 0.0013<br>pH = 7.4	FW = 0.945<br>NL = 0.0023<br>NP = 0.0013<br>pH = 7.4	[53,56,57]

EW: Extracellular Water, **IW:** Intracellular Water, **NL:** Neutral Lipids, **NP:** Neutral Phospholipids, **AP:** Acidic Phospholipids, **AR:** Albumin ratio, **LR:** lipoprotein ratio

Table S2. Physiological parameters - Human PBPK model

Parameter	Equation / Value		Reference
Hematocrit	Male: 0.43, CV (%): 6.5<br>Female: 0.38, CV (%): 7.1		[52]
Human Serum Albumin (g/L)	45		[52]
Height	Male: $175.32 + 0.1113 * age - 0.0025 * age^2$<br>CV (%): 3.9<br>Female: $161.66 + 0.1319 * age - 0.0027 * age^2$<br>CV (%): 3.9		[28,52]
Weight	Male: $\exp(2.643 + 0.0099 * Height)$<br>CV (%): 15<br>Female: $\exp(2.7383 + 0.0091 * Height)$<br>CV (%): 18.8		[28,52]
Body Surface Area	$0.007184 * Height$^$0.725 * Weight$^0.425		[28,52]
**Volumes (L)**			
**Parameter**	**Equation / Value**	**Notes**	**Reference**
**Other Organ Compartments:**			
Vlung	Male: $((29.08 * (Height/100) * Weight$^$0.5 + 11.06 + 35.47 * (Height/100) * Weight$^$0.5 + 5.53)/1000)/1.05$<br>Female: $((31.46 * (Height/100) * Weight$^$0.5 + 1.43 + 35.3 * (Height/100) * Weight$^$0.5 + 1.53)/1000)/1.05$		[28,52]
Vadipose	Male: $(1.36 * Weight)/(Height/100) - 42$<br>Female: $(1.61 * Weight)/(Height/100) - 38.3$		[28,52]
Vbone	Male: $((Weight - Vadipose * 0.92)/1.3 * 0.058)$<br>Female: $((Weight - Vadipose * 0.92)/1.3 * 0.051)$		[28,52]
Vheart	Male: $((155.18 * (BSA)$^$1.29)/1000)/1.05$<br>Female: $((124.13 * (BSA)$^$1.242)/1000)/1.05$		[28,52]
Vkidney	$((15.4 + 2.04 * Weight + 51.8 * (Height/100)$^$2)/1000)/1.05$		[28,52]
Vmuscle	Male: $(0.244 * Weight + 7.8 * (Height/100) - 0.098 * age + 3.3)/1.04$<br>Female: $(0.244 * Weight + 7.8 * (Height/100) - 0.098 * age - 3.3)/1.04$		[28,52]
Vskin	$(BSA/1000) * 45.655 + (BSA/1000) * 1240$	Skin thickness was derived from SimCYP simulator	[28,52]
Vspleen	$(6.516 * Weight$^$0.797)/1000$		[28,52]
Vgut	Male: $0.021 * (Weight - Vadipose * 0.92)/1.05$<br>Female: $0.027 * (Weight - Vadipose * 0.92)/1.05$		[28,52]
Vliver	$(1072.8 * (BSA) - 345.7)/1000$		[28,52]
Vbla	Male:<br>$(((13.1 * Height + 18.05 * Weight - 480)/0.5723)/1000)/2$<br>Female:<br>$(((35.5 * Height + 2.27 * Weight - 3382)/0.6178)/1000)/2$		[28,52]
Vblv	Male:<br>$(((13.1 * Height + 18.05 * Weight - 480)/0.5723)/1000)/2$		[28,52]

	Female: $(((35.5 * Height + 2.27 * Weight - 3382)/0.6178)/1000)/2$		
Vrest	$Weight - Vblv - Vbla - Vliver - Vgut - Vspleen - Vskin - Vmuscle - Vkidney - Vheart - Vbone - Vadipose - Vlung - Vbrain$	Body density of 1 kg/L assumed	-
**Brain Compartments:**			
Vbrain total	$(1.449 – 3.62/BW)/1.04$		[28,52]
Vbrain blood	$0.05 * Vbraintotal$		[28,52]
Vccsf	Male: $Vbrainmale * 0.105 * 0.8$<br>Female: $Vbrainfemale * 0.092 * 0.8$		[28,52]
Vscsf	Male: $Vbrainmale * 0.105 * 0.2$<br>Female: $Vbrainfemale * 0.092 * 0.2$	Vscsf capped at 20% of the total csf	[28,52]
Vendothelial mass	$Vbrainmale * 0.005$		[28,52]
Vbrain mass	$Vbrain - Vbrainendo - Vbb - Vccsf - Vscsf$		[28,52]
**Fluid flow rates (L/hr)**			
**Parameter**	**Equation / Value**	**Notes**	**Reference**
**Other organ Compartments:**			
Qcarout	$BSA * 60 * (3 - 0.01 * (age - 20))$	Cardiac output * fractional tissue flow	[28,52]
Qlung	$Qcarout * 1$		[28,52]
Qadipose	Male: $Qcarout * 0.05$<br>Female: $Qcarout * 0.085$		[28,52]
Qbone	$Qcarout * 0.05$		[28,52]
Qheart	Male: $Qcarout * 0.04$<br>Female: $Qcarout * 0.05$		[28,52]
Qkidney	Male: $Qcarout * 0.19$<br>Female: $Qcarout * 0.17$		[28,52]
Qmuscle	Male: $Qcarout * 0.17$<br>Female: $Qcarout * 0.12$		[28,52]
Qskin	$Qcarout * 0.05$		[28,52]
Qspleen	Male: $Qcarout * 0.02$<br>Female: $Qcarout * 0.03$		[28,52]
Qgut	Male: $Qcarout * 0.15$<br>Female: $Qcarout * 0.17$		[28,52]
Qha	$Qcarout * 0.065$		[28,52]
Qliver	Male: $Qcarout * 0.235$<br>Female: $Qcarout * 0.265$		[28,52]
Qrestflow	Male: $Qcarout * 0.095$<br>Female: $Qcarout * 0.09$	Qcarout – organ flows	[28,52]
**Brain Compartment:**			
Qbrain	$Qcarout * 0.12$	Cardiac output * fractional tissue flow	[52,59]
Qproductionrate	0.021, CV (%): 10		[52,59]
Qbulk	$0.25 * Qproductionrate$, CV (%): 8	Assumed: Relative CSF flows (as part of Qcsfproduction rate)	[52,59]
Qssink	$0.38 * (0.75 * Qproductionrate + Qbulk)$, CV (%): 30		[52,59]
Qsout	$0.9 * Qssink$, CV (%): 100%		[52,59]
Qsin	$(Qssink + Qsout)$		[52,59]
Qcsink	$(0.75 * Qproductionrate + Qbulk - Qsin + Qsout)$		[52,59]
**Tissue Composition (fraction)**			

**Parameter**	**Rowdgers and Rowland**	**Poulin and Theil**	**Berezhkovskiy**	**Reference**
Adipose	EW = 0.141<br>IW = 0.039<br>NL = 0.79<br>NP = 0.002<br>AP = 0.4<br>AR = 0.037<br>LR = 0.068	FW = 0.18<br>NL = 0.79<br>NP = 0.002<br>pH = 7	FW = 0.18<br>NL = 0.79<br>NP = 0.002<br>pH = 7	[53,56,57]
Bone	EW = 0.098<br>IW = 0.341<br>NL = 0.074<br>NP = 0.0011<br>AP = 0.67<br>AR = 0.1<br>LR = 0.05	FW = 0.439<br>NL = 0.074<br>NP = 0.0011<br>pH = 7	FW = 0.439<br>NL = 0.074<br>NP = 0.0011<br>pH = 7	[53,56,57]
Gut	EW = 0.267<br>IW = 0.451<br>NL = 0.0487<br>NP = 0.0163<br>AP = 2.84<br>AR = 0.158<br>LR = 0.141	FW = 0.718<br>NL = 0.0487<br>NP = 0.0163<br>pH = 7	FW = 0.718<br>NL = 0.0487<br>NP = 0.0163<br>pH = 7	[53,56,57]
Heart	EW = 0.313<br>IW = 0.445<br>NL = 0.0115<br>NP = 0.0166<br>AP = 3.07<br>AR = 0.157<br>LR = 0.16	FW = 0.758<br>NL = 0.0115<br>NP = 0.0166<br>pH = 7	FW = 0.758<br>NL = 0.0115<br>NP = 0.0166<br>pH = 7	[53,56,57]
Kidney	EW = 0.283<br>IW = 0.50<br>NL = 0.0207<br>NP = 0.0162<br>AP = 2.48<br>AR = 0.13<br>LR = 0.137	FW = 0.783<br>NL = 0.0207<br>NP = 0.0162<br>pH = 7.2	FW = 0.783<br>NL = 0.0207<br>NP = 0.0162<br>pH = 7.2	[53,56,57]
Liver	EW = 0.165<br>IW = 0.586<br>NL = 0.0348<br>NP = 0.0252<br>AP = 5.09<br>AR = 0.086<br>LR = 0.161	FW = 0.751<br>NL = 0.0348<br>NP = 0.0252<br>pH = 7	FW = 0.751<br>NL = 0.0348<br>NP = 0.0252<br>pH = 7	[53,56,57]
Lung	EW = 0.348<br>IW = 0.463<br>NL = 0.003<br>NP = 0.009<br>AP = 0.5<br>AR = 0.212<br>LR = 0.168	FW = 0.811<br>NL = 0.003<br>NP = 0.009<br>pH = 6.7	FW = 0.811<br>NL = 0.003<br>NP = 0.009<br>pH = 6.7	[53,56,57]
Muscle	EW = 0.091<br>IW = 0.669<br>NL = 0.0238<br>NP = 0.0072<br>AP = 2.49<br>AR = 0.034<br>LR = 0.059	FW = 0.76<br>NL = 0.0238<br>NP = 0.0072<br>pH = 7	FW = 0.76<br>NL = 0.0238<br>NP = 0.0072<br>pH = 7	[53,56,57]
Skin	EW = 0.623<br>IW = 0.0947<br>NL = 0.0284	FW = 0.7177<br>NL = 0.0284<br>NP = 0.0111	FW = 0.7177<br>NL = 0.0284<br>NP = 0.0111	[53,56,57]

	NP = 0.0111<br>AP = 1.32<br>AR = 0.277<br>LR = 0.096	pH = 7	pH = 7	
Spleen	EW = 0.208<br>IW = 0.579<br>NL = 0.0201<br>NP = 0.0198<br>AP = 2.81<br>AR = 0.097<br>LR = 0.207	FW = 0.787<br>NL = 0.0201<br>NP = 0.0198<br>pH = 7	FW = 0.787<br>NL = 0.0201<br>NP = 0.0198<br>pH = 7	[53,56,57]
Plasma	EW = 0.945<br>IW = 0<br>NL = 0.0035<br>NP = 0.0023<br>AP = 0.04<br>AR = NA<br>LR = NA	FW = 0.945<br>NL = 0.0035<br>NP = 0.0023<br>pH = 7.4	FW = 0.945<br>NL = 0.0035<br>NP = 0.0023<br>pH = 7.4	[53,56,57]

EW: Extracellular Water, **IW:** Intracellular Water, **NL:** Neutral Lipids, **NP:** Neutral Phospholipids, **AP:** Acidic Phospholipids, **AR:** Albumin ratio, **LR:** lipoprotein ratio

Table S3. Physicochemical and Drug Specific Parameters for Acetaminophen, Oxycodone, Lacosamide, Ibuprofen & Levetiracetam.

Drug		Acetaminophen Parameter	Ref.	Oxycodone Parameter	Ref.	Lacosamide Parameter	Ref.	Ibuprofen Parameter	Ref.	Levetiracetam Parameter	Ref.
Molecular weight (g/mol)		151.2	[28,59]	315.4	[31]	250.3	[31]	206.285	[92]	170.21	[93]
Log P		0.46	[28,59]	1.2	[31]	0.3	[31]	3.97	[92]	-0.67	[94]
Pka		9.38	[28,59]	8.53	[31]	12.4	[31]	4.91	[92]	15.74	[94]
Compound Type		Monoprotic acid	[28,59]	Monoprotic base	[31]	Neutral	[31]	Monoprotic acid	[92]	Neutral	[58]
Kp Method		Rodgers & Rowland [59]		Poulin & Theil		Rodgers & Rowland [31]		Berezhkovskiy		Rodgers & Rowland	
BCS Classification		III	[95]	I or III	[96]	I	[97]	II	[98]	I	[99]
CL - Human (L/hr)		22.8 CV(%):35.4	[59]	49.2 CV (%):30	[31]	2.13 CV (%):30	[31]	3.88 CV (%):30	[38]	3.96 CV(%):42	[93]
CL-scalar		-	-	0.8	#	0.8	#	-	-	-	-
BP	Rats	1	[100]	1.3	[8]	1	[42]	0.55	[101]	1.05	[34]
	Humans	1.09	[28,59]	1.3	[31]	1	[31]	1	[31]	0.85	[102]
Fupl	Rats	0.88	[100]	0.56	[8]	0.96	[42]	0.04	[103]	0.9-1	[104]
	Humans	0.855	[28,59]	0.59	[31]	1	[31]	(0.00567+ 0.0107)/2	[105,106]	0.95	[104]
Fubm	Rats	0.83	[107]	0.39	[8]	0.899	[108]	0.3117 - Assumed same as human		0.98	[46]
	Humans	0.747	[107]	0.45	[8]	0.899 - Assumed same as rats		0.3117	[109]	0.98	γ
Fuccsf		1	*	1	*	1	*	1	*	1	*
Fuscsf		1	**	1	**	1	**	1	**	1	**
P_{BBB} (cm/sec)		11.11×10^{-6}	#	26.79×10^{-6}	#	18.8×10^{-6}	#	35.138×10^{-6}	#	1.5295×10^{-6}	#
PS_B	Rats (ml/min)	0.179	#	0.434	#	0.305	#	0.569	#	0.02456	#
	Humans (L/hr)	5.98	#	14.47	#	10.15	#	18.97	#	0.8187	#
PS_E (Brain-CCSF)	Rats (ml/min)	80	##	80	##	80	##	80	##	80	##
	Humans (L/hr)	300	##	300	##	300	##	300	##	300	##
PSc (BCSFB)	Rats (ml/min)	0.0179	[59], β	0.0434	[59], β	0.0305	[59], β	0.0569	[59], β	0.002456	[59], β
	Humans (L/hr)	0.598	[59], β	1.447	[59], β	1.015	[59], β	1.897	[59], β	0.08187	[59], β

PS_B, PS_C and PS_E: permeability surface area products between brain blood and brain mass, brain blood and cranial CSF respectively; **Fubm, Fupl, Fuccsf, Fuscsf**: Fraction unbound in brain mass, plasma, cranial CSF and spinal CSF respectively; **CL:** clearance; **BP:** Blood to plasma ratio; **BCS:** Biopharmaceutical classification system

γ Assumed (low conc. of protein than plasma)
* Assumed (low conc. of protein in Cranial-CSF);
** Assumed (low conc. of protein in Spinal-CSF);
# Optimized using Rat PBPK Model / Human PBPK Model,
Assumed to be no barrier
β Assumed to be 1/10th of PS_B.
