# Supplementary material for: Interspecies Brain PBPK Modeling Platform to Predict Passive Transport through the Blood–Brain Barrier and Assess Target Site Disposition
Source: Pharmaceutics. 2024 Feb 4;16(2):226. doi: 10.3390/pharmaceutics16020226 (PMC10892872; doi:10.3390/pharmaceutics16020226)
Supplement: Supplementary file 1 [file pharmaceutics-16-00226-s001.zip › Supplementary_Model Code - Rats & Humans.pdf]

Article

Interspecies Brain PBPK Modeling Platform to Predict Passive Transport through the Blood-Brain Barrier and Assess Target-Site Disposition

Supplementary Materials

Parsshava Mehta [1], Amira Soliman [1,2], Leyanis Rodriguez-Vera [3], Stephan Schmidt [1], Paula Muniz [3], Monica Rodriguez [3], Marta Forcadell [4], Emili Gonzalez-Perez [4] and Valvanera Vozmediano [1,3*]

[1] Center for Pharmacometrics and Systems Pharmacology, Department of Pharmaceutics, College of Pharmacy, University of Florida, Orlando, FL 32827, USA; parsshava.mehta@ufl.edu (P.M.); amira.soliman@ufl.edu (A.S.); sschmidt@cop.ufl.edu (S.S.)
[2] Department of Pharmacy Practice, Faculty of Pharmacy, Helwan University, Helwan 11795, Egypt
[3] Model Informed Development, CTI Laboratories, Covington, KY 41011, USA; lrodriguez@ctifacts.com (L.R.-V.); pmuniz@ctifacts.com (P.M.); mrodriguez@ctifacts.com (M.R.)
[4] Neuraxpharm Pharmaceuticals SL, Clinical Research and Evidence-Generation Science, 08970 Barcelona, Spain; forcadellmarta@gmail.com (M.F.); emiligonzalezperez@gmail.com (E.G.-P.)
* Correspondence: valva@ufl.edu or vvozmediano@ctifacts.com

Julia Code for PBPK Model in Rats

```
## PBPK model in Rats – Drug  (Example Code – Not for any specific drug)

## Load necessary libraries
```

```
using Random, Pumas, PumasUtilities, CairoMakie, PumasPlots, Serialization, GlobalSensitivity, Random, Weave, CSV, Latexify, Chain, HTTP, AlgebraOfGraphics, DataFramesMeta, CategoricalArrays

## Drug Specific Parameters ## ---------------------------------------------------------------------------

   Molecular_Weight = ___

  ##Blood to Plasma Ratio
   BP_rat      = ___                # Blood to Plasma Ratio

  ##Unboud Fraction of drug
   Fubm_rat   = ___                 # Fraction unbound in brain mass
   Fupl_rat    = ___                # Fraction unbound in plasma
   Fubb_rat   =  Fupl_rat/BP_rat    # Fraction unbound in blood
   Fuccsf_rat  = ___                # Fraction unbound in cranial CSF
   Fuscsf_rat  = ___                # Fraction unbound in spinal CSF

  ##Physico Chemical Properties
   LogP    = ___
   pKa     = ___
   Type_of_compound = ___           # 1-Neutral, 2-Monoprotic Acid, 3-Monoprotic Base

  ##Calculation of KP Methods

   ## Rodgers and Rowland
```

```
rat_RR = CSV.File("/home/jrun/data/code/UF_LabLesvi/Data_IN/Kp_Methods/rat_RR.csv",
        missingstring=["", "NA", "."])
rat_RR_df = DataFrame(rat_RR)

function calcKp_RR(logP, pKa, fup, BP, type, P_ow_calc, dat::DataFrame)

 dat_all  = dat[findall(in(["Bone","Gut","Heart","Kidney","Liver","Lung","Muscle","Pancreas","Skin","Spleen"]), dat.tissue), :] #df except
Adipose, RBC and Plasma
 dat_ad   = filter(row -> row.tissue == ("Adipose"), dat)     #df for Adipose
 dat_rbc  = filter(row -> row.tissue == ("RBCs"), dat)        #df for RBCs
 dat_plas = filter(row -> row.tissue == ("Plasma"), dat)      #df for Plasma

 pH_IW  = 6.9       #pH of intracellular tissue water
 pH_P   = 7.4       #pH of plasma
 pH_RBC = 7.27      #pH of blood cells
 P      = 10^(logP)   #Octanol:water partition coefficient

 #logP_OW calculation, Oil:Water partition coefficient
 if P_ow_calc == 1
  logP_OW = 1.115*logP - 1.35     #Hansch Equation
 else
  logP_OW = -4.653+((7.972/(1+10^(0.1175-0.2849*logP))))  #Bartels et al
 end

 P_OW = 10^(logP_OW)
```

```
#Ka   = 10^(-pKa)
HCT  = 0.439           #Hematocrit

Kpu_bc = (HCT - 1 + BP)/(HCT*fup)

#Calculate X value
if type==1         #Neutral
  X = 0
elseif type==2     #Monoprotic Acid
  X = 10^(pH_IW-pKa)
elseif type==3     #Monoprotic Base
  X = 10^(pKa-pH_IW)
elseif type==4     #Diprotic Acid
  X = 10^(pH_IW-pKa[1])+10^(2*pH_IW-pKa[1]-pKa[2])
elseif type==5     #Diprotic Base
  X = 10^(pKa[2]-pH_IW)+10^(pKa[1]+pKa[2]-2*pH_IW)
elseif type==6     #Monoprotic acid and Monoprotic base (acid comes first)
  X = 10^(pKa[2]-pH_IW)+10^(pH_IW-pKa[1])
elseif type==7     #Triprotic Acid
  X = 10^(pH_IW-pKa[1])+10^(2*pH_IW-pKa[1]-pKa[2])+10^(3*pH_IW-pKa[1]-pKa[2]-pKa[3])
elseif type==8     #Triprotic Base
  X = 10^(pKa[3]-pH_IW)+10^(pKa[3]+pKa[2]-2*pH_IW)+10^(pKa[1]+pKa[2]+pKa[3]-3*pH_IW)
elseif type==9     #Diprotic acid and Monoprotic base (first two are acid)
  X = 10^(pKa[3]-pH_IW)+10^(pH_IW-pKa[1])+10^(2*pH_IW-pKa[1]-pKa[2])
```

```
elseif type==10    #Monoprotic acid and Diprotic base (first one is acid)
  X = 10^(pH_IW-pKa[1])+10^(pKa[3]-pH_IW)+10^(pKa[2]+pKa[3]-2*pH_IW)
else
  X = "Type of Compound Not specified Correctly"
end

#Calculate Y value
if type==1         #Neutral
  Y = 0
elseif type==2     #Monoprotic Acid
  Y = 10^(pH_P-pKa)
elseif type==3     #Monoprotic Base
  Y = 10^(pKa-pH_P)
elseif type==4     #Diprotic Acid
  Y = 10^(pH_P-pKa[1])+10^(2*pH_P-pKa[1]-pKa[2])
elseif type==5     #Diprotic Base
  Y = 10^(pKa[2]-pH_P)+10^(pKa[1]+pKa[2]-2*pH_P)
elseif type==6     #Monoprotic acid and Monoprotic base (acid comes first)
  Y = 10^(pKa[2]-pH_P)+10^(pH_P-pKa[1])
elseif type==7     #Triprotic Acid
  Y = 10^(pH_P-pKa[1])+10^(2*pH_P-pKa[1]-pKa[2])+10^(3*pH_P-pKa[1]-pKa[2]-pKa[3])
elseif type==8     #Triprotic Base
  Y = 10^(pKa[3]-pH_P)+10^(pKa[3]+pka[2]-2*pH_P)+10^(pKa[1]+pKa[2]+pKa[3]-3*pH_P)
elseif type==9     #Diprotic acid and Monoprotic base (first two are acid)
```

```
  Y = 10^(pKa[3]-pH_P)+10^(pH_P-pKa[1])+10^(2*pH_P-pKa[1]-pKa[2])
elseif type==10    #Monoprotic acid and Diprotic base (first one is acid)
  Y = 10^(pH_P-pKa[1])+10^(pKa[3]-pH_P)+10^(pKa[2]+pKa[3]-2*pH_P)
else
  Y = "Type of Compound Not specified Correctly"
end

#Calculate Z value
if type==1         #Neutral
  Z = 1
elseif type==2     #Monoprotic Acid
  Z = 1
elseif type==3     #Monoprotic Base
  Z = 10^(pKa-pH_RBC)
elseif type==4     #Diprotic Acid
  Z = 1
elseif type==5     #Diprotic Base
  Z = 10^(pKa[2]-pH_RBC)+10^(pKa[1]+pKa[2]-2*pH_RBC)
elseif type==6     #Monoprotic acid and Monoprotic base (acid comes first)
  Z = 10^(pKa[2]-pH_RBC)+10^(pH_RBC-pKa[1])
elseif type==7     #Triprotic Acid
  Z = 1
elseif type==8     #Triprotic Base
  Z = 10^(pKa[3]-pH_RBC)+10^(pKa[3]+pka[2]-2*pH_RBC)+10^(pKa[1]+pKa[2]+pKa[3]-3*pH_RBC)
```

```
elseif type==9     #Diprotic acid and Monoprotic base (first two are acid)
  Z = 10^(pKa[3]-pH_RBC)+10^(pH_RBC-pKa[1])+10^(2*pH_RBC-pKa[1]-pKa[2])
elseif type==10    #Monoprotic acid and Diprotic base (first one is acid)
  Z = 10^(pH_RBC-pKa[1])+10^(pKa[3]-pH_RBC)+10^(pKa[2]+pKa[3]-2*pH_RBC)
else
  Z = "Type of Compound Not specified Correctly"
end

Ka_AP = (Kpu_bc - (1 + Z)/(1 + Y)*dat_rbc[1,:f_iw] - (P*dat_rbc[1,:f_n_l] + (0.3*P + 0.7)*dat_rbc[1,:f_n_pl])/(1 + Y)) * (1 + Y)/dat_rbc[1,:f_a_pl]/Z
Ka_PR = ((1/fup) - 1 - ((P*dat_plas[1,:f_n_l] + (0.3*P + 0.7)*dat_plas[1,:f_n_pl])/(1+Y)))

# Assign the neutral type_calc=3, moderate to strong bases type_calc=1 and everything else type_calc=2
if type==1
  type_calc = 3
elseif (type==3 && pKa>7)
  type_calc = 1
elseif (type==5 && pKa>7)
  type_calc = 1
elseif (type==6 && pKa[2] > 7)
  type_calc = 1
elseif (type==8 && pKa[1] > 7)
  type_calc = 1
elseif (type==9 && pKa[3]>7)
  type_calc = 1
```

```
elseif (type==10 && pKa[2]>7)
  type_calc = 1
else
  type_calc = 2
end

# Multiply by fup to get Kp rather than Kpu
if (type_calc==1)  #moderate to strong bases
  Kp_all = (dat_all[!,:f_ew] + ((1 + X)/(1 + Y))*dat_all[!,:f_iw] + ((P*dat_all[!,:f_n_l] + (0.3*P + 0.7)*dat_all[!,:f_n_pl]))/(1 + Y) + (Ka_AP*dat_all[!,:f_a_pl]*X)/(1 + Y))*fup       #non lipid
  Kp_ad = (dat_ad[!, :f_ew] + ((1 + X)/(1 + Y))*dat_ad[!, :f_iw] + ((P_OW*dat_ad[!, :f_n_l] + (0.3*P_OW + 0.7)*dat_ad[!, :f_n_pl]))/(1 + Y) + (Ka_AP*dat_ad[!, :f_a_pl]*X)/(1 + Y))*fup  #lipid
elseif (type_calc==2) #acidic and zwitterions
  Kp_all = (dat_all[!, :f_ew] + ((1 + X)/(1 + Y))*dat_all[!, :f_iw] + ((P*dat_all[!, :f_n_l] + (0.3*P + 0.7)*dat_all[!, :f_n_pl]))/(1 + Y) + (Ka_PR*dat_all[!, :AR]))*fup  #non lipid
  Kp_ad = (dat_ad[!, :f_ew] + ((1 + X)/(1 + Y))*dat_ad[!, :f_iw] + ((P_OW*dat_ad[!, :f_n_l] + (0.3*P_OW + 0.7)*dat_ad[!, :f_n_pl]))/(1 + Y) + (Ka_PR*dat_ad[!, :AR]))*fup  #lipid
else  #neutrals
  Kp_all = (dat_all[!, :f_ew] + ((1 + X)/(1 + Y))*dat_all[!, :f_iw] + ((P*dat_all[!, :f_n_l] + (0.3*P + 0.7)*dat_all[!, :f_n_pl]))/(1 + Y) + (Ka_PR*dat_all[!, :LR]))*fup  #non lipid
  Kp_ad = (dat_ad[!, :f_ew] + ((1 + X)/(1 + Y))*dat_ad[!, :f_iw] + ((P_OW*dat_ad[!, :f_n_l] + (0.3*P_OW + 0.7)*dat_ad[!, :f_n_pl]))/(1 + Y) + (Ka_PR*dat_ad[!, :LR]))*fup  #lipid
end

Kp = [Kp_ad; Kp_all]
Kp_df = DataFrame.(Kp_values = Kp)
Kp_tissue1 = transform(dat_all, :tissue => ByRow(x-> lowercase(x)) => :tissue)
```

```
  Kp_tissue2 = "Kp" .* select(Kp_tissue1, :tissue)
  Kp_adipose = DataFrame.(tissue = ["Kpadipose"])

  Kp_tissue = outerjoin(Kp_adipose, Kp_tissue2, on = :tissue)

  Kp_values = hcat(Kp_tissue, Kp_df)

  return(Kp_values)
end

## Poulin and Theil
rat_PT = CSV.File("/home/jrun/data/code/UF_LabLesvi/Data_IN/Kp_Methods/rat_PT_Edited.csv",
        missingstring=["", "NA", "."])
rat_PT_df = DataFrame(rat_PT)

function calcKp_PT(logP, pKa, fup, type, dat)

  dat_all  = dat[findall(in(["Bone","Gut","Heart","Kidney","Liver","Lung","Muscle","Spleen","Skin"]), dat.tissue), :]  #df for all except
Adipose and Plasma
  dat_ad   = filter(row -> row.tissue == ("Adipose"), dat)  #df for Adipose
  dat_plas = filter(row -> row.tissue == ("Plasma"), dat)   #df for Plasma

  Vwp  = dat_plas[1,:f_water]
  Vnlp = dat_plas[1,:f_n_l]
  Vphp = dat_plas[1,:f_pl]
```

```
Vwt   = dat_all[!, :f_water]
Vwad  = dat_ad[1,:f_water]
Vnlt  = dat_all[!,:f_n_l]
Vnlad = dat_ad[1,:f_n_l]
Vpht  = dat_all[!,:f_pl]
Vphad = dat_ad[1,:f_pl]

pH = dat_ad[1,:pH]

logD = 1.115*logP-1.35 #logD is the olive oil:buffer(water) partition coefficient of nonionized species

#Calculate logD_star value
if type==1          #Neutral
  logD_star = logD
elseif type==2      #Monoprotic Acid
  logD_star = logD-log10(1+10^(pH-pKa))
elseif type==3      #Monoprotic Base
  logD_star = logD-log10(1+10^(pKa-pH))
elseif type==4      #Diprotic Acid
  logD_star = logD-log10(1+10^(2*pH-pKa[1]-pKa[2]))
elseif type==5      #Diprotic Base
  logD_star = logD-log10(1+10^(pKa[1]+pKa[2]-2*pH))
elseif type==6      #Monoprotic acid and Monoprotic base (acid comes first)
```

```
  logD_star = logD-log10(1+10^(pKa[2]-pKa[1]))
elseif type==7     #Triprotic Acid
  logD_star = logD-log10(1+10^(3*pH-pKa[1]-pKa[2]-pKa[3]))
elseif type==8     #Triprotic Base
  logD_star = logD-log10(1+10^(pKa[1]+pKa[2]+pKa[3]-3*pH))
elseif type==9     #Diprotic acid and Monoprotic base (first two are acid)
  logD_star = logD-log10(1+10^(pH-pKa[1]-pKa[2]+pKa[3]))
elseif type==10    #Monoprotic acid and Diprotic base (first one is acid)
  logD_star = logD-log10(1+10^(pKa[2]+pKa[3]-pKa[1]-pH))
else
  logD_star = "Type of Compound Not specified Correctly"
end

D_star = 10^logD_star
Kpad   = ((D_star*(Vnlad+0.3*Vphad)+(1*(Vwad+0.7*Vphad)))/(D_star*(Vnlp+0.3*Vphp)+(1*(Vwp+0.7*Vphp)))) * fup

P = 10^logP
fut = 1/(1+((1-fup)/fup)*0.5)

Kpt = ((P*(Vnlt+0.3*Vpht)+(1*(Vwt+0.7*Vpht)))/(P*(Vnlp+0.3*Vphp)+(1*(Vwp+0.7*Vphp)))) * (fup/fut)

Kp         = [Kpad; Kpt]
Kp_df      = DataFrame.(Kp_values = Kp)
Kp_tissue1 = transform(dat_all, :tissue => ByRow(x-> lowercase(x)) => :tissue)
```

```
        Kp_tissue2 = "Kp" .* select(Kp_tissue1, :tissue)
        Kp_adipose = DataFrame.(tissue = ["Kpadipose"])

        Kp_tissue  = outerjoin(Kp_adipose, Kp_tissue2, on = :tissue)

        Kp_values  = hcat(Kp_tissue, Kp_df)
        push!(Kp_values,["Kppancreas", 0])

        return(Kp_values)
      end

    ##Berezhkovisky

      rat_Berez = CSV.File("/home/jrun/data/code/UF_LabLesvi/Data_IN/Kp_Methods/rat_Berez_Edited.csv",
                        missingstring=["", "NA", "."])
      rat_Berez_df = DataFrame(rat_Berez)

      function calcKp_Berez(logP, pKa, fup, type, dat)

        dat_all  = dat[findall(in(["Bone","Gut","Heart","Kidney","Liver","Lung","Muscle","Spleen","Skin"]), dat.tissue), :]  #df for all except
Adipose and Plasma
        dat_ad   = filter(row -> row.tissue == ("Adipose"), dat)  #df for Adipose
        dat_plas = filter(row -> row.tissue == ("Plasma"), dat)   #df for Plasma

        Vwp  = dat_plas[1,:f_water]
```

```
Vnlp = dat_plas[1,:f_n_l]
Vphp = dat_plas[1,:f_pl]

Vwt   = dat_all[!, :f_water]
Vwad  = dat_ad[1,:f_water]
Vnlt  = dat_all[!,:f_n_l]
Vnlad = dat_ad[1,:f_n_l]
Vpht  = dat_all[!,:f_pl]
Vphad = dat_ad[1,:f_pl]

pH = dat_ad[1,:pH]

logD = 1.115*logP-1.35 #logD is the olive oil:buffer(water) partition coefficient of nonionized species

#Calculate logD_star value
if type==1         #Neutral
  logD_star = logD
elseif type==2     #Monoprotic Acid
  logD_star = logD-log10(1+10^(pH-pKa))
elseif type==3     #Monoprotic Base
  logD_star = logD-log10(1+10^(pKa-pH))
elseif type==4     #Diprotic Acid
  logD_star = logD-log10(1+10^(2*pH-pKa[1]-pKa[2]))
elseif type==5     #Diprotic Base
```

```
  logD_star = logD-log10(1+10^(pKa[1]+pKa[2]-2*pH))
elseif type==6     #Monoprotic acid and Monoprotic base (acid comes first)
  logD_star = logD-log10(1+10^(pKa[2]-pKa[1]))
elseif type==7     #Triprotic Acid
  logD_star = logD-log10(1+10^(3*pH-pKa[1]-pKa[2]-pKa[3]))
elseif type==8     #Triprotic Base
  logD_star = logD-log10(1+10^(pKa[1]+pKa[2]+pKa[3]-3*pH))
elseif type==9     #Diprotic acid and Monoprotic base (first two are acid)
  logD_star = logD-log10(1+10^(pH-pKa[1]-pKa[2]+pKa[3]))
elseif type==10    #Monoprotic acid and Diprotic base (first one is acid)
  logD_star = logD-log10(1+10^(pKa[2]+pKa[3]-pKa[1]-pH))
else
  logD_star = "Type of Compound Not specified Correctly"
end

D_star = 10^logD_star
Kpad   = ((D_star*(Vnlad+0.3*Vphad)+((Vwad+0.7*Vphad)))/(D_star*(Vnlp+0.3*Vphp)+((Vwp/fup)+0.7*Vphp)))

P   = 10^logP
fut = 1/(1+((1-fup)/(2*fup)))

Kpt = ((P*(Vnlt+0.3*Vpht)+((Vwt/fut)+0.7*Vpht))/(P*(Vnlp+0.3*Vphp)+((Vwp/fup)+0.7*Vphp)))

Kp        = [Kpad; Kpt]
```

```
    Kp_df      = DataFrame.(Kp_values = Kp)
    Kp_tissue1 = transform(dat_all, :tissue => ByRow(x-> lowercase(x)) => :tissue)
    Kp_tissue2 = "Kp" .* select(Kp_tissue1, :tissue)
    Kp_adipose = DataFrame.(tissue = ["Kpadipose"])

    Kp_tissue  = outerjoin(Kp_adipose, Kp_tissue2, on = :tissue)

    Kp_values  = hcat(Kp_tissue, Kp_df)
    push!(Kp_values,["Kppancreas", 0])

    return(Kp_values)
  end
```

```
## Calculation of the Kp values using the function for each
  R_R   = calcKp_RR(LogP, pKa, Fubb_rat, BP_rat, Type_of_compound, 1, rat_RR_df)
  P_T   = calcKp_PT(LogP, pKa, Fubb_rat, Type_of_compound, rat_PT_df)
  Berez = calcKp_Berez(LogP, pKa, Fubb_rat, Type_of_compound, rat_Berez_df)

  df_Kp_values = outerjoin(R_R, P_T, Berez, on= :tissue, makeunique=true)
  rename!(df_Kp_values, [:tissue, :R_R, :P_T, :Berez])

  ##Select the best KP Method and add the variable in the Final Kp Method
```

```
    Best_Kp        = ___
    convert_to_kpu = 1  #Divide by unbound fraction if you want to calculate unbound Kp (Kpu), else set to 1
    Kp_scalar      = 1

    Kpadipose_rat = (only(Best_Kp[in.(Best_Kp.tissue, Ref(["Kpadipose"])), :Kp_values])  / convert_to_kpu ) * Kp_scalar
    Kpbone_rat    = (only(Best_Kp[in.(Best_Kp.tissue, Ref(["Kpbone"])), :Kp_values]) / convert_to_kpu ) * Kp_scalar
    Kpgut_rat     = (only(Best_Kp[in.(Best_Kp.tissue, Ref(["Kpgut"])), :Kp_values])  / convert_to_kpu ) * Kp_scalar
    Kpheart_rat   = (only(Best_Kp[in.(Best_Kp.tissue, Ref(["Kpheart"])), :Kp_values])  / convert_to_kpu ) * Kp_scalar
    Kpkidney_rat  = (only(Best_Kp[in.(Best_Kp.tissue, Ref(["Kpkidney"])), :Kp_values])  / convert_to_kpu ) * Kp_scalar
    Kpliver_rat   = (only(Best_Kp[in.(Best_Kp.tissue, Ref(["Kpliver"])), :Kp_values])  / convert_to_kpu ) * Kp_scalar
    Kplung_rat    = (only(Best_Kp[in.(Best_Kp.tissue, Ref(["Kplung"])), :Kp_values])  / convert_to_kpu ) * Kp_scalar
    Kpmuscle_rat  = (only(Best_Kp[in.(Best_Kp.tissue, Ref(["Kpmuscle"])), :Kp_values])  / convert_to_kpu ) * Kp_scalar
    Kpskin_rat    = (only(Best_Kp[in.(Best_Kp.tissue, Ref(["Kpskin"])), :Kp_values])  / convert_to_kpu ) * Kp_scalar
    Kpspleen_rat  = (only(Best_Kp[in.(Best_Kp.tissue, Ref(["Kpspleen"])), :Kp_values])  / convert_to_kpu ) * Kp_scalar
    Kprest_rat     = ((Kpadipose_rat +Kplung_rat +Kpbone_rat +Kpheart_rat +Kpkidney_rat +Kpmuscle_rat
                     +Kpskin_rat +Kpspleen_rat +Kpgut_rat +Kpliver_rat )/10)


## Physiology of Rat

Weight_of_rat = 250   #gms (this will help to scale all volumes and blood flows accordingly)

## PBPK Model Rats ## ----------------------------------------------------------------------------------------
```

```
full_pbpk_model_rat_v1 = @model begin
  @param begin
    tvCL        ∈ RealDomain(lower = 0.1, upper=30.0)
    tvP_bbb     ∈ RealDomain(lower=1.67*10^-9, upper=1.67*10^-4)
    σ₁          ∈ RealDomain(lower = 0.001)
    σ₂          ∈ RealDomain(lower = 0.001)
  end

  @pre begin

    Weight   = Weight_of_rat      #All parameters are scaled based on Weight of Rat

  ## Volumes (mL)  ## ----------------------------------------------------------------------------------------------

    ## Brain
    Vbtotal  = 1.24*(Weight/250)        #Vtotal is scaled to weight of rat since it is used in calculation of brain compartments

    Vbb      = Vbtotal*0.0310               #Brain blood (mL)
    Vccsf    = Vbtotal*0.144                #Cranial CSF (mL) (LV + TFV)
    Vscsf    = Vccsf*0.18750                #Spinal CSF (mL)  (CM + SAS)
    Vbm      = Vbtotal - Vccsf - Vscsf   #Brain mass (mL)
    Vecf     = 0.29 *(Weight/250)          #Exctracellular Fluid, Part of Brain Mass
```

```
## Other Organ Volumes
Vlung    = 1.24*(Weight/250)            #Lung (mL)
Vadipose = 16.66*(Weight/250)           #Adipose (mL)
Vbone    = 15.68*(Weight/250)           #Bone (mL)
Vheart   = 1.05*(Weight/250)            #Heart (mL)
Vkidney  = 2.19*(Weight/250)            #Kidney (mL)
Vmuscle  = 116.13*(Weight/250)          #Muscle (mL)
Vskin    = 39.41*(Weight/250)           #Skin (mL)
Vspleen  = 0.57*(Weight/250)            #Spleen (mL)
Vgut     = 6.19*(Weight/250)            #Gut (mL)
Vliver   = 8.57*(Weight/250)            #Liver (mL)
Vbla     = 6.8*(Weight/250)             #Arterial (mL)
Vblv     = 13.6*(Weight/250)            #Venous (mL)
Vrest    = 1.3*(Weight/250)             #Rest of the organs (mL)

## Tissue Blood/CSF flows (mL/min) ## ----------------------------------------------------------------------

Qcarout  = 80*(Weight/250)              #Cardiac output (mL/min)

## Brain
Qbrain         = 1.12*(Weight/250)              #Bloodflow to brain (mL/min)

Qproductionrate = 0.0026*(Weight/250)           #CSF production rate (mL/min)
Qbulk          = 0.085*Qproductionrate          #bulk flow from brain to CSF (mL/min)
```

```
Qssink        = 0.37*(0.75*Qproductionrate+Qbulk)     #flow spinal CSF to blood (mL/min)
Qsout         = 0.9*Qssink                            #flow from spinal CSF to cranial CSF (mL/min)
Qsin          = (Qssink+Qsout)                        #flow from cranial CSF to spinal CSF (mL/min)
Qcsink        = (0.75*Qproductionrate + Qbulk - Qsin + Qsout)  #flow from cranial CSF to blood (mL/min)


## Other organs
Qadipose      = 4.72*(Weight/250)       #Adipose Tissue (mL/min)
Qbone         = 8.08*(Weight/250)       #Bone (mL/min)
Qheart        = 3.2*(Weight/250)        #Heart (mL/min)
Qkidney       = 11.6*(Weight/250)       #Kidney (mL/min)
Qmuscle       = 18.96*(Weight/250)    #Muscle (mL/min)
Qskin         = 4.08*(Weight/250)       #Skin (mL/min)
Qspleen       = 0.88*(Weight/250)      #Spleen (mL/min)
Qgut          = 8.08*(Weight/250)       #Gut (mL/min)  [Stomach & Oesophagus, Small Intestine, Large Intestine]
Qha           = 1.28*(Weight/250)       #Hepatic Artery (mL/min)
Qliver        = 10.24*(Weight/250)    #Liver (mL/min)
Qrest         = 1*(Weight/250)          #Pancreas (mL/min)
Qlung         = 80*(Weight/250)       #Lung (mL/min) [100% of cardiac Output]

## Drug Specific Parameters ## ------------------------------------------------------------------------------------


MW      = Molecular_Weight
BP      = BP_rat
Fubm    = Fubm_rat              # Fraction unbound in brain mass
```

```
Fupl    = Fupl_rat                    # Fraction unbound in plasma
Fubb    = Fubb_rat                    # Fraction unbound in blood
Fuccsf  = Fuccsf_rat                  # Fraction unbound in cranial CSF
Fuscsf  = Fuscsf_rat                  # Fraction unbound in spinal CSF

Kpadipose = Kpadipose_rat
Kplung    = Kplung_rat
Kpbone    = Kpbone_rat
Kpheart   = Kpheart_rat
Kpkidney  = Kpkidney_rat
Kpmuscle  = Kpmuscle_rat
Kpskin    = Kpskin_rat
Kpspleen  = Kpspleen_rat
Kpgut     = Kpgut_rat
Kpliver   = Kpliver_rat
Kprest    = Kprest_rat

# Permeability surface area product (mL/min)
Permeability_bbb = tvP_bbb                    #cm/sec
BBB_surface_area = 1.8*150                    #cm^2

PSb = Permeability_bbb*BBB_surface_area*60    # PS BBB , Permeability surface area product (mL/min) ->  Convert from cm^3/sec to
mL/min
PSe = 80                                      # PS brainmass-cranial CSF
PSc = 0.1 * PSb                               # PS BCSFB , BBB Surface area of CSF is 1/10th of Brain
```

```
## PK Parameters ## ---------------------------------------------------------------------------------------------------

  CL = tvCL

end

 @dynamics begin

  #### Differential Equations are in terms of Concentrations ####

  Cbb'    = (Qbrain*(Cbla-Cbb) + PSb * (Fubm*Cbm - Fubb * Cbb) + PSc*(Fuccsf * Cccsf - Fubb * Cbb)+ Qssink*Cscsf + Qcsink*Cccsf)/Vbb

  Cbm'    = (PSb * (Fubb * Cbb - Fubm * Cbm) + PSe*(Fuccsf*Cccsf-Fubm*Cbm) - Qbulk*Cbm)/Vbm

  Cccsf'  = ((PSe * (Fubm*Cbm -Fuccsf*Cccsf)) + PSc * (Fubb*Cbb - Fuccsf*Cccsf) + Qbulk*Cbm + Qsout*Cscsf - Qsin * Cccsf - Qcsink * Cccsf)/ Vccsf

  Cscsf'  = (Qsin*Cccsf - Qsout * Cscsf - Qssink*Cscsf)/Vscsf


  Cblv'   = (Qadipose*(Cadipose/Kpadipose*BP) + Qbone*(Cbone/Kpbone*BP) + Qheart*(Cheart/Kpheart*BP) + Qkidney*(Ckidney/Kpkidney*BP) + Qmuscle*(Cmuscle/Kpmuscle*BP) + Qskin* (Cskin/Kpskin*BP) + Qliver*(Cliver/Kpliver*BP) + Qbrain*(Cbb) + Qrest*(Crest/Kprest*BP) - Qlung*(Cblv) -(CL*Cblv))/Vblv

  Cbla'   = (Qlung*(Clung/Kplung*BP) - Qrest*Cbla - Qbrain*Cbla - Qadipose*Cbla - Qbone*Cbla - Qheart*Cbla - Qkidney*Cbla - Qmuscle*Cbla - Qskin*Cbla - Qspleen*Cbla - Qgut*Cbla - Qha*Cbla)/Vbla

  Clung'  = (Qlung*(Cblv-(Clung/Kplung*BP)))/Vlung

  Cadipose' = (Qadipose*(Cbla-(Cadipose/Kpadipose*BP)))/Vadipose

  Cbone'  = (Qbone*(Cbla-(Cbone/Kpbone*BP)))/Vbone

  Cheart' = (Qheart*(Cbla-(Cheart/Kpheart*BP)))/Vheart
```

```
    Ckidney'  = (Qkidney*(Cbla-(Ckidney/Kpkidney*BP)))/Vkidney
    Cmuscle'  = (Qmuscle*(Cbla-(Cmuscle/Kpmuscle*BP)))/Vmuscle
    Cskin'    = (Qskin*(Cbla-(Cskin/Kpskin*BP)))/Vskin
    Cspleen'  = (Qspleen*(Cbla-(Cspleen/Kpspleen*BP)))/Vspleen
    Crest'    = (Qrest*(Cbla-(Crest/Kprest*BP)))/Vrest
    Cgut'     = (Qgut*(Cbla-(Cgut/Kpgut*BP)))/Vgut
    Cliver'   = (Qha*Cbla + Qgut*(Cgut/Kpgut*BP) + Qspleen*(Cspleen/Kpspleen*BP) - Qliver*(Cliver/Kpliver*BP))/Vliver

  end

  @derived begin
    "Plasma Concentrations (ug/mL)"
    Cpplasma          = @. Cblv
    plasma_ug_mL    ~ @. Normal(Cpplasma, σ₁)

    "Brain Concentrations (ug/mL)"
    Cbrainmass       = @. Cbm
    brain_ug_mL     ~ @. Normal(Cbrainmass, σ₂)
  end
end

## Initial Parameters for the drug

initial_param =   (   tvCL     = ___,
```

```
                    tvP_bbb  = ___,
                    σ1       = ___,
                    σ2       = ___)

## Import Dataset of Rat for Drug (Author)
Drug_rat_data_df = DataFrame(CSV.File("/home/jrun/data/code/UF_LabLesvi/Data_IN/Drug/Author_Drug.csv",
                    missingstring=["", "NA", "."]))


## Read the dataset into Pumas
Drug_rat = read_pumas(Drug_rat_data_df,
                    id           = :ID,
                    time         = :TIME_MINS,
                    amt          = :AMT,
                    observations = [:plasma_ug_mL, :brain_ug_mL],
                    evid         = :EVID,
                    cmt          = :CMT,
                    rate         = :RATE)


## Fit the data using a Naive Pooled Approach
pkfit_np = fit(full_pbpk_model_rat_v1,
                    Drug_rat,
                    initial_param,
```

```
                    Pumas.NaivePooled())
```

Julia Code for PBPK Model in Humans

```
##PBPK model in Humans - Drug

## Load necessary libraries
using Random, Pumas, CairoMakie, PumasPlots, GlobalSensitivity, Random, Weave, CSV, Latexify, Chain, HTTP, AlgebraOfGraphics,
DataFramesMeta, CategoricalArrays

## Number of individuals, the code is written for both males (N) and females (NF) to account for physiological differences between gender
   N = ___
   NF = ___
   Total_Samples = N + NF

## Age (y)
  Random.seed!(123)
  agemultiplemale=rand(Uniform(___,___), N)       #age (range) multiple virtual male individuals (uniform distribution)
  Random.seed!(1234)
  agemultiplefemale=rand(Uniform(___,___), NF)      #age (range) multiple virtual female individuals (uniform distribution)
```

```
## Height (cm), Weight (kg) and BSA (m^2) Calculations: (equations are derived from SimCYP adult simulator)

 #### Males:
   Height_male_adults_gr18=175.32 .+ 0.1113 .* agemultiplemale .- 0.0025 .* agemultiplemale .^2
   Random.seed!(123)
   Height_male_adults_gr18cv=rand(Normal(0,0.039),N) # CV
   Height_male_adults_gr18var= Height_male_adults_gr18 .* exp.(Height_male_adults_gr18cv)

   Weight_male_adults_gr18=exp.(2.643 .+ 0.0099 .* Height_male_adults_gr18var)
   Random.seed!(123)
  Weight_male_adults_gr18cv=rand(Normal(0,0.15),N) # CV
  Weight_male_adults_gr18var= Weight_male_adults_gr18 .* exp.(Weight_male_adults_gr18cv)

  BSA_male_adults_gr18=0.007184 .* Height_male_adults_gr18var .^ 0.725 .*Weight_male_adults_gr18var .^ 0.425

 #### Females:
   Height_female_adults_gr18=161.66 .+ 0.1319 .* agemultiplefemale .- 0.0027 .* agemultiplefemale .^2
   Random.seed!(123)
   Height_female_adults_gr18cv=rand(Normal(0,0.039),NF) # CV
   Height_female_adults_gr18var= Height_female_adults_gr18 .* exp.(Height_female_adults_gr18cv)

   Weight_female_adults_gr18=exp.(2.7383 .+ 0.0091 .* Height_female_adults_gr18var)
   Random.seed!(123)
   Weight_female_adults_gr18cv=rand(Normal(0,0.188),NF)
   Weight_female_adults_gr18var= Weight_female_adults_gr18 .* exp.(Weight_female_adults_gr18cv)
```

```
    BSA_female_adults_gr18=0.007184 .* Height_female_adults_gr18var .^0.725 .* Weight_female_adults_gr18var .^0.425

    Weight_male_females = [Weight_male_adults_gr18var;Weight_female_adults_gr18var]

#### Parameters ####

## Volumes (L)  ## ----------------------------------------------------------------------------------------------------

  #### Male
    Vbrainmale=(1.449 .- 3.62 ./ Weight_male_adults_gr18var) ./ 1.04        #brain total
    Vbbmale=0.05 .* Vbrainmale                                              #brain blood
    Vccsfmale= Vbrainmale .* 0.105 .* 0.8                                   #cranial CSF
    Vscsfmale=Vbrainmale .* 0.105 .* 0.2                                    #spinal CSF
    Vbrainendomale=Vbrainmale .* 0.005                                      #endothelial cells
    Vbmmale=Vbrainmale .- Vbrainendomale .- Vbbmale .- Vccsfmale .- Vscsfmale #brain mass

    Vlungmale=((29.08 .* (Height_male_adults_gr18var ./ 100) .* Weight_male_adults_gr18var .^ 0.5 .+ 11.06 .+ 35.47 .*
(Height_male_adults_gr18var ./ 100) .* Weight_male_adults_gr18var .^0.5 .+ 5.53) ./ 1000) ./ 1.05

    Vadiposemale=(1.36 .* Weight_male_adults_gr18var) ./ (Height_male_adults_gr18var ./ 100) .- 42
    Vadiposemale[Vadiposemale .< 0] .= 0.01    #used to avoid negative values

    Vbonemale= (Weight_male_adults_gr18var .- Vadiposemale .* 0.92) ./ 1.3 .* 0.058
    Vbonemale[Vbonemale .< 0] .= 0.01         #used to avoid negative values

    Vheartmale=(155.18 .* (BSA_male_adults_gr18) .^1.29) ./ 1000 ./ 1.05
```

```
Vkidneymale=((15.4 .+ (2.04 .* Weight_male_adults_gr18var) .+ 51.8 .* (Height_male_adults_gr18var ./ 100) .^2) ./ 1000) ./ 1.05
Vmusclemale=((0.244 .* Weight_male_adults_gr18var)
        .+ (7.8 .* (Height_male_adults_gr18var ./ 100))
        .- (0.098 .* agemultiplemale) .+ 3.3) ./ 1.04
Vskinmale= (((BSA_male_adults_gr18 ./ 1000) .* 45.655)
        .+ ((BSA_male_adults_gr18 ./ 1000) .* 1240))
Vspleenmale=(6.516 .* (Weight_male_adults_gr18var) .^ 0.797) ./ 1000
Vgutmale=(0.021 .* (Weight_male_adults_gr18var .- Vadiposemale .* 0.92)) ./ 1.05
Vlivermale=((1072.8 .* (BSA_male_adults_gr18)) .- 345.7) ./ 1000
Vblamale=((((13.1 .* Height_male_adults_gr18var)
        .+ (18.05 .* Weight_male_adults_gr18var) .- 480) ./ 0.5723) ./ 1000) ./ 2
Vblvmale=((((13.1 .* Height_male_adults_gr18var) .+ (18.05 .* Weight_male_adults_gr18var) .- 480) ./ 0.5723) ./1000) ./ 2

Vrestmale=(Weight_male_adults_gr18var .- Vblvmale .- Vblamale .- Vlivermale
        .- Vgutmale .- Vspleenmale .- Vskinmale .- Vmusclemale .- Vkidneymale
        .- Vheartmale .- Vbonemale .- Vadiposemale .- Vlungmale .- Vbrainmale) #rest tissue
Vrestmale[Vrestmale .< 0] .= 0.01     #used to avoid negative values

#### Female
Vbrainfemale=(1.449 .- 3.62 ./ Weight_female_adults_gr18var) ./ 1.04          #brain total
Vbbfemale=0.05 .* Vbrainfemale                                                #brain blood
Vccsffemale=Vbrainfemale .* 0.092 .* 0.8                                      #cranial CSF
Vscsffemale=Vbrainfemale .* 0.092 .* 0.2                                      #spinal CSF
Vbrainendofemale=Vbrainfemale .* 0.005                                        #endothelial cells
Vbmfemale=Vbrainfemale .- Vbrainendofemale .- Vbbfemale .- Vccsffemale .- Vscsffemale #brain mass
```

```
Vlungfemale=((31.46 .* (Height_female_adults_gr18var ./ 100)
        .* Weight_female_adults_gr18var .^ 0.5 .+ 1.43 .+ 35.3
        .*(Height_female_adults_gr18var ./ 100)
        .* Weight_female_adults_gr18var .^ 0.5 .+ 1.53) ./ 1000) ./ 1.05

Vadiposefemale=(1.61 .* Weight_female_adults_gr18var) ./ (Height_female_adults_gr18var ./ 100) .- 38.3
Vadiposefemale[Vadiposefemale .< 0] .= 0.01      #used to avoid negative values

Vbonefemale= (Weight_female_adults_gr18var .- Vadiposefemale .* 0.92) ./ 1.3 .* 0.051
Vbonefemale[Vbonefemale .< 0] .= 0.01      #used to avoid negative values

Vheartfemale=(124.13 .* (BSA_female_adults_gr18) .^ 1.242) ./ 1000 ./ 1.05
Vkidneyfemale=((15.4 .+ (2.04 .* Weight_female_adults_gr18var) .+ 51.8
         .* (Height_female_adults_gr18var ./ 100) .^ 2) ./ 1000) ./ 1.05
Vmusclefemale=((0.244 .* Weight_female_adults_gr18var)
         .+ (7.8 .* (Height_female_adults_gr18var ./ 100))
         .- (0.098 .* agemultiplefemale) .- 3.3) ./ 1.04
Vskinfemale=((BSA_female_adults_gr18 ./ 1000) .* 45.655) .+ ((BSA_female_adults_gr18 ./ 1000) .* 1240)
Vspleenfemale=(6.516 .* (Weight_female_adults_gr18var) .^ 0.797) ./ 1000
Vgutfemale=(0.027 .* (Weight_female_adults_gr18var .- Vadiposefemale .* 0.92)) ./ 1.05
Vliverfemale=((1072.8 .* (BSA_female_adults_gr18)) .- 345.7) ./ 1000
Vblafemale=((((35.5 .* Height_female_adults_gr18var) .+ (2.27
      .* Weight_female_adults_gr18var) .- 3382) ./ 0.6178) ./ 1000) ./ 2
Vblvfemale=((((35.5 .* Height_female_adults_gr18var)
      .+ (2.27 .* Weight_female_adults_gr18var) .- 3382) ./ 0.6178) ./ 1000) ./ 2
```

```
Vrestfemale= Weight_female_adults_gr18var .- Vblvfemale .- Vblafemale .-
        Vliverfemale .- Vgutfemale .- Vspleenfemale .- Vskinfemale .-
        Vmusclefemale .- Vkidneyfemale .- Vheartfemale .- Vbonefemale .-
        Vadiposefemale .- Vlungfemale .- Vbrainfemale #rest tissue
Vrestfemale[Vrestfemale .< 0] .= 0.01     #used to avoid negative values

## Tissue Blood/CSF flows (L/h) ## ---------------------------------------------------------------------------------

#### Male

##Blood flows in Brain Compartments

Qcaroutmale= BSA_male_adults_gr18 .* 60 .* (3 .- (0.01 .* (agemultiplemale .- 20))) #cardiac output

Qbrainmale=Qcaroutmale .* 0.12 #blood flow to brain blood

Qproductionratemale=0.021 #CSF production
Random.seed!(123)
QproductionrateCVmale=rand(Normal(0,0.1),N)
Q_productionratemale= Qproductionratemale .* exp.(QproductionrateCVmale)

Qbulkmale=0.25 .* Q_productionratemale #bulk flow from brain mass to Cranial CSF
Random.seed!(123)
QbulkCVmale=rand(Normal(0,0.08),N)
Q_bulkmale= Qbulkmale .* exp.(QbulkCVmale)
```

```
Qssinkmale=0.38 .* (0.75 .* Q_productionratemale .+ Q_bulkmale) #flow from Spinal CSF to brain blood
Random.seed!(123)
QssinkCVmale=rand(Normal(0,0.3),N)
Q_ssinkmale = Qssinkmale .* exp.(QssinkCVmale)

Qsoutmale=0.9 .* Q_ssinkmale #flow from Spinal CSF to Cranial CSF
Random.seed!(123)
QsoutCVmale=rand(Normal(0,1),N)
Q_soutmale = Qsoutmale .* exp.(QsoutCVmale)

Qsinmale = Q_ssinkmale .+ Q_soutmale #flow from Cranial CSF to Spinal CSF

Qcsinkmale= (0.75 .* Q_productionratemale .+ Q_bulkmale .- Qsinmale .+ Q_soutmale) #flow from Cranial CSF to brain blood

##Blood flows in Other Compartments of the Body

Qadiposemale=Qcaroutmale .* 0.05
Qbonemale=Qcaroutmale .* 0.05
Qheartmale=Qcaroutmale .* 0.04
Qkidneymale=Qcaroutmale .* 0.19
Qmusclemale=Qcaroutmale .* 0.17
Qskinmale=Qcaroutmale .* 0.05
Qspleenmale=Qcaroutmale .* 0.02
Qgutmale=Qcaroutmale .* 0.15
Qhamale=Qcaroutmale .* 0.065
Qlivermale=Qcaroutmale .* 0.235
```

```
Qlungmale=Qcaroutmale .* 1
Qrestmale=Qcaroutmale .* 0.095     #Blood Flow to 'rest' of the organs not included as compartments
Qrestmale[Qrestmale .< 0] .= 0.1   #included to set negative restflow to 0.1

#### Female

##Blood flows in Brain Compartments

Qcaroutfemale= BSA_female_adults_gr18 .* 60 .* (3 .- (0.01 .* (agemultiplefemale .- 20))) #cardiac output

Qbrainfemale=Qcaroutfemale .* 0.12 #blood flow to brain blood

Qproductionratefemale=0.021 #CSF production
Random.seed!(123)
QproductionrateCVfemale=rand(Normal(0,0.1),NF)
Q_productionratefemale= Qproductionratefemale .* exp.(QproductionrateCVfemale)

Qbulkfemale=0.25 .* Q_productionratefemale #bulk flow from brain mass to Cranial CSF
Random.seed!(123)
QbulkCVfemale=rand(Normal(0,0.08),NF)
Q_bulkfemale= Qbulkfemale .* exp.(QbulkCVfemale)

Qssinkfemale=0.38 .* (0.75 .* Q_productionratefemale .+ Q_bulkfemale) #flow from Spinal CSF to brain blood
Random.seed!(123)
QssinkCVfemale=rand(Normal(0,0.3),NF)
Q_ssinkfemale = Qssinkfemale .* exp.(QssinkCVfemale)
```

```
Qsoutfemale=0.9 .* Q_ssinkfemale #flow from Spinal CSF to Cranial CSF
Random.seed!(123)
QsoutCVfemale=rand(Normal(0,1),NF)
Q_soutfemale = Qsoutfemale .* exp.(QsoutCVfemale)

Qsinfemale = Q_ssinkfemale .+ Q_soutfemale #flow from Cranial CSF to Spinal CSF

Qcsinkfemale= (0.75 .* Q_productionratefemale .+ Q_bulkfemale .- Qsinfemale .+ Q_soutfemale) #flow from Cranial CSF to brain blood

##Blood flows in Other Compartments of the Body

Qadiposefemale=Qcaroutfemale .* 0.085
Qbonefemale=Qcaroutfemale .* 0.05
Qheartfemale=Qcaroutfemale .* 0.05
Qkidneyfemale=Qcaroutfemale .* 0.17
Qmusclefemale=Qcaroutfemale .* 0.12
Qskinfemale=Qcaroutfemale .* 0.05
Qspleenfemale=Qcaroutfemale .* 0.03
Qgutfemale=Qcaroutfemale .* 0.17
Qhafemale=Qcaroutfemale .* 0.065
Qliverfemale=Qcaroutfemale .* 0.265
Qlungfemale=Qcaroutfemale .* 1
Qrestfemale=Qcaroutfemale .* 0.09      #Blood Flow to 'rest' of the organs not included as compartments
Qrestfemale[Qrestfemale .< 0] .= 0.1   #included to set negative restflow to 0.1
```

```
## Combine Parameters of Males and Females together for Simulation

    Qbrain_1   = [Qbrainmale;Qbrainfemale]
    Qbulk_1    = [Qbulkmale;Qbulkfemale]
    Qssink_1   = [Q_ssinkmale;Q_ssinkfemale]
    Qcsink_1   = [Qcsinkmale;Qcsinkfemale]
    Qsout_1    = [Q_soutmale;Q_soutfemale]
    Qsin_1     = [Qsinmale;Qsinfemale]

    Qadipose_1 = [Qadiposemale;Qadiposefemale]   #Adipose Tissue (L/hr)
    Qbone_1    = [Qbonemale;Qbonefemale]           #Bone (L/hr)
    Qheart_1   = [Qheartmale;Qheartfemale]         #Heart (L/hr)
    Qkidney_1  = [Qkidneymale;Qkidneyfemale]       #Kidney (L/hr)
    Qmuscle_1  = [Qmusclemale;Qmusclefemale]       #Muscle (L/hr)
    Qskin_1    = [Qskinmale;Qskinfemale]           #Skin (L/hr)
    Qspleen_1  = [Qspleenmale;Qspleenfemale]       #Spleen (L/hr)
    Qgut_1     = [Qgutmale;Qgutfemale]             #Gut (L/hr)  [Stomach & Oesophagus, Small Intestine, Large Intestine]
    Qha_1      = [Qhamale;Qhafemale]               #Hepatic Artery (L/hr)
    Qliver_1   = [Qlivermale;Qliverfemale]         #Liver (L/hr)
    Qrest_1    = [Qrestmale;Qrestfemale]           #Pancreas (L/hr)
    Qlung_1    = [Qlungmale;Qlungfemale]           #Lung (L/hr) [100% of cardiac Output]

    ## Volumes of organs (L)
    Vbb_1   = [Vbbmale;Vbbfemale]          #Brain blood (L)
    Vccsf_1 = [Vccsfmale;Vccsffemale]     #Cranial CSF (L)
    Vscsf_1 = [Vscsfmale;Vscsffemale]     #Spinal CSF (L)
```

```
Vbm_1   = [Vbmmale;Vbmfemale]      #Brain mass (L)

Vecf_1  = 0.29   #Exctracellular Fluid, Part of Brain Mass (L)

Vlung_1    = [Vlungmale;Vlungfemale]                #Lung (L)
Vadipose_1 = [Vadiposemale;Vadiposefemale]    #Adipose (L)
Vbone_1    = [Vbonemale;Vbonefemale]              #Bone (L)
Vheart_1   = [Vheartmale;Vheartfemale]            #Heart (L)
Vkidney_1  = [Vkidneymale;Vkidneyfemale]      #Kidney (L)
Vmuscle_1  = [Vmusclemale;Vmusclefemale]     #Muscle (L)
Vskin_1    = [Vskinmale;Vskinfemale]               #Skin (L)
Vspleen_1  = [Vspleenmale;Vspleenfemale]       #Spleen (L)
Vgut_1     = [Vgutmale;Vgutfemale]                  #Gut (L)
Vliver_1   = [Vlivermale;Vliverfemale]             #Liver (L)
Vbla_1     = [Vblamale;Vblafemale]                  #Arterial (L)
Vblv_1     = [Vblvmale;Vblvfemale]                  #Venous (L)
Vrest_1    = [Vrestmale;Vrestfemale]                #Rest of the organs (L)

#Adding the Volumes and Blood Flows as covariates for each subject

choose_covariates(x) = (;
                       Qbrain_human    = Qbrain_1[x],
                       Qbulk_human     = Qbulk_1[x],
                       Qssink_human    = Qssink_1[x],
                       Qcsink_human    = Qcsink_1[x],
                       Qsout_human     = Qsout_1[x],
```

```
Qsin_human      = Qsin_1[x],
Qadipose_human  = Qadipose_1[x],
Qbone_human     = Qbone_1[x],
Qheart_human    = Qheart_1[x],
Qkidney_human   = Qkidney_1[x],
Qmuscle_human   = Qmuscle_1[x],
Qskin_human     = Qskin_1[x],
Qspleen_human   = Qspleen_1[x],
Qgut_human      = Qgut_1[x],
Qha_human       = Qha_1[x],
Qliver_human    = Qliver_1[x],
Qrest_human     = Qrest_1[x],
Qlung_human     = Qlung_1[x],
Vbb_human       = Vbb_1[x],
Vccsf_human     = Vccsf_1[x],
Vscsf_human     = Vscsf_1[x],
Vbm_human       = Vbm_1[x],
Vlung_human     = Vlung_1[x],
Vadipose_human  = Vadipose_1[x],
Vbone_human     = Vbone_1[x],
Vheart_human    = Vheart_1[x],
Vkidney_human   = Vkidney_1[x],
Vmuscle_human   = Vmuscle_1[x],
Vskin_human     = Vskin_1[x],
Vspleen_human   = Vspleen_1[x],
Vgut_human      = Vgut_1[x],
```

```
                 Vliver_human    = Vliver_1[x],
                 Vbla_human      = Vbla_1[x],
                 Vblv_human      = Vblv_1[x],
                 Vrest_human     = Vrest_1[x])

## Drug Specific Parameters ## -------------------------------------------------------------------------------------

 ##Blood to Plasma Ratio
  BP_human      = ___                  # Blood to Plasma Ratio

 ##Unboud Fraction of drug
  Fubm_human    = ___                                   # Fraction unbound in brain mass
  Fupl_human    = ___                                   # Fraction unbound in plasma
  Fubb_human    = Fupl_human/BP_human          # Fraction unbound in blood
  Fuccsf_human  = ___                                  # Fraction unbound in cranial CSF
  Fuscsf_human  = ___                                  # Fraction unbound in spinal CSF

 ##Physico Chemical Properties
  LogP    = ___
  pKa     = ___
  Type_of_compound = ___              # 1-Neutral, 2-Monoprotic Acid, 3-Monoprotic Base

 ##Calculation of KP Methods

  ## Rodgers and Rowland
   human_RR = CSV.File("/home/jrun/data/code/UF_LabLesvi/Data_IN/Kp_Methods/human_RR.csv",
```

```
            missingstring=["", "NA", "."])
    human_RR_df = DataFrame(human_RR)

    function calcKp_RR(logP, pKa, fup, BP, type, P_ow_calc, dat::DataFrame)

      dat_all  = dat[findall(in(["Bone","Gut","Heart","Kidney","Liver","Lung","Muscle","Pancreas","Skin","Spleen"]), dat.tissue), :] #df except
Adipose, RBC and Plasma
      dat_ad   = filter(row -> row.tissue == ("Adipose"), dat)       #df for Adipose
      dat_rbc  = filter(row -> row.tissue == ("RBCs"), dat)          #df for RBCs
      dat_plas = filter(row -> row.tissue == ("Plasma"), dat)        #df for Plasma

      pH_IW  = 7          #pH of intracellular tissue water
      pH_P   = 7.4        #pH of plasma
      pH_RBC = 7.22       #pH of blood cells
      P      = 10^(logP)  #Octanol:water partition coefficient

      #logP_OW calculation, Oil:Water partition coefficient
      if P_ow_calc == 1
        logP_OW = 1.115*logP - 1.35      #Hansch Equation
      else
        logP_OW = -4.653+((7.972/(1+10^(0.1175-0.2849*logP))))  #Bartels et al
      end

      P_OW = 10^(logP_OW)
      #Ka   = 10^(-pKa)
      HCT  = 0.45         #Hematocrit
```

```
Kpu_bc = (HCT - 1 + BP)/(HCT*fup)

#Calculate X value
if type==1        #Neutral
  X = 0
elseif type==2    #Monoprotic Acid
  X = 10^(pH_IW-pKa)
elseif type==3    #Monoprotic Base
  X = 10^(pKa-pH_IW)
elseif type==4    #Diprotic Acid
  X = 10^(pH_IW-pKa[1])+10^(2*pH_IW-pKa[1]-pKa[2])
elseif type==5    #Diprotic Base
  X = 10^(pKa[2]-pH_IW)+10^(pKa[1]+pKa[2]-2*pH_IW)
elseif type==6    #Monoprotic acid and Monoprotic base (acid comes first)
  X = 10^(pKa[2]-pH_IW)+10^(pH_IW-pKa[1])
elseif type==7    #Triprotic Acid
  X = 10^(pH_IW-pKa[1])+10^(2*pH_IW-pKa[1]-pKa[2])+10^(3*pH_IW-pKa[1]-pKa[2]-pKa[3])
elseif type==8    #Triprotic Base
  X = 10^(pKa[3]-pH_IW)+10^(pKa[3]+pKa[2]-2*pH_IW)+10^(pKa[1]+pKa[2]+pKa[3]-3*pH_IW)
elseif type==9    #Diprotic acid and Monoprotic base (first two are acid)
  X = 10^(pKa[3]-pH_IW)+10^(pH_IW-pKa[1])+10^(2*pH_IW-pKa[1]-pKa[2])
elseif type==10   #Monoprotic acid and Diprotic base (first one is acid)
  X = 10^(pH_IW-pKa[1])+10^(pKa[3]-pH_IW)+10^(pKa[2]+pKa[3]-2*pH_IW)
else
  X = "Type of Compound Not specified Correctly"
```

```
end

#Calculate Y value
if type==1        #Neutral
  Y = 0
elseif type==2     #Monoprotic Acid
  Y = 10^(pH_P-pKa)
elseif type==3     #Monoprotic Base
  Y = 10^(pKa-pH_P)
elseif type==4     #Diprotic Acid
  Y = 10^(pH_P-pKa[1])+10^(2*pH_P-pKa[1]-pKa[2])
elseif type==5     #Diprotic Base
  Y = 10^(pKa[2]-pH_P)+10^(pKa[1]+pKa[2]-2*pH_P)
elseif type==6     #Monoprotic acid and Monoprotic base (acid comes first)
  Y = 10^(pKa[2]-pH_P)+10^(pH_P-pKa[1])
elseif type==7     #Triprotic Acid
  Y = 10^(pH_P-pKa[1])+10^(2*pH_P-pKa[1]-pKa[2])+10^(3*pH_P-pKa[1]-pKa[2]-pKa[3])
elseif type==8     #Triprotic Base
  Y = 10^(pKa[3]-pH_P)+10^(pKa[3]+pka[2]-2*pH_P)+10^(pKa[1]+pKa[2]+pKa[3]-3*pH_P)
elseif type==9     #Diprotic acid and Monoprotic base (first two are acid)
  Y = 10^(pKa[3]-pH_P)+10^(pH_P-pKa[1])+10^(2*pH_P-pKa[1]-pKa[2])
elseif type==10    #Monoprotic acid and Diprotic base (first one is acid)
  Y = 10^(pH_P-pKa[1])+10^(pKa[3]-pH_P)+10^(pKa[2]+pKa[3]-2*pH_P)
else
  Y = "Type of Compound Not specified Correctly"
end
```

```
#Calculate Z value
if type==1        #Neutral
  Z = 1
elseif type==2     #Monoprotic Acid
  Z = 1
elseif type==3     #Monoprotic Base
  Z = 10^(pKa-pH_RBC)
elseif type==4     #Diprotic Acid
  Z = 1
elseif type==5     #Diprotic Base
  Z = 10^(pKa[2]-pH_RBC)+10^(pKa[1]+pKa[2]-2*pH_RBC)
elseif type==6     #Monoprotic acid and Monoprotic base (acid comes first)
  Z = 10^(pKa[2]-pH_RBC)+10^(pH_RBC-pKa[1])
elseif type==7     #Triprotic Acid
  Z = 1
elseif type==8     #Triprotic Base
  Z = 10^(pKa[3]-pH_RBC)+10^(pKa[3]+pka[2]-2*pH_RBC)+10^(pKa[1]+pKa[2]+pKa[3]-3*pH_RBC)
elseif type==9     #Diprotic acid and Monoprotic base (first two are acid)
  Z = 10^(pKa[3]-pH_RBC)+10^(pH_RBC-pKa[1])+10^(2*pH_RBC-pKa[1]-pKa[2])
elseif type==10    #Monoprotic acid and Diprotic base (first one is acid)
  Z = 10^(pH_RBC-pKa[1])+10^(pKa[3]-pH_RBC)+10^(pKa[2]+pKa[3]-2*pH_RBC)
else
  Z = "Type of Compound Not specified Correctly"
end
```

```
Ka_AP = (Kpu_bc - (1 + Z)/(1 + Y)*dat_rbc[1,:f_iw] - (P*dat_rbc[1,:f_n_l] + (0.3*P + 0.7)*dat_rbc[1,:f_n_pl])/(1 + Y)) * (1 + Y)/dat_rbc[1,:f_a_pl]/Z

Ka_PR = ((1/fup) - 1 - ((P*dat_plas[1,:f_n_l] + (0.3*P + 0.7)*dat_plas[1,:f_n_pl])/(1+Y)))


# Assign the neutral type_calc=3, moderate to strong bases type_calc=1 and everything else type_calc=2
if type==1
  type_calc = 3
elseif (type==3 && pKa>7)
  type_calc = 1
elseif (type==5 && pKa>7)
  type_calc = 1
elseif (type==6 && pKa[2] > 7)
  type_calc = 1
elseif (type==8 && pKa[1] > 7)
  type_calc = 1
elseif (type==9 && pKa[3]>7)
  type_calc = 1
elseif (type==10 && pKa[2]>7)
  type_calc = 1
else
  type_calc = 2
end


# Multiply by fup to get Kp rather than Kpu
if (type_calc==1)  #moderate to strong bases
```

```
    Kp_all = (dat_all[!,:f_ew] + ((1 + X)/(1 + Y))*dat_all[!,:f_iw] + ((P*dat_all[!,:f_n_l] + (0.3*P + 0.7)*dat_all[!,:f_n_pl]))/(1 + Y) + (Ka_AP*dat_all[!,:f_a_pl]*X)/(1 + Y))*fup       #non lipid

    Kp_ad = (dat_ad[!, :f_ew] + ((1 + X)/(1 + Y))*dat_ad[!, :f_iw] + ((P_OW*dat_ad[!, :f_n_l] + (0.3*P_OW + 0.7)*dat_ad[!, :f_n_pl]))/(1 + Y) + (Ka_AP*dat_ad[!, :f_a_pl]*X)/(1 + Y))*fup  #lipid

  elseif (type_calc==2) #acidic and zwitterions

    Kp_all = (dat_all[!, :f_ew] + ((1 + X)/(1 + Y))*dat_all[!, :f_iw] + ((P*dat_all[!, :f_n_l] + (0.3*P + 0.7)*dat_all[!, :f_n_pl]))/(1 + Y) + (Ka_PR*dat_all[!, :AR]))*fup  #non lipid

    Kp_ad = (dat_ad[!, :f_ew] + ((1 + X)/(1 + Y))*dat_ad[!, :f_iw] + ((P_OW*dat_ad[!, :f_n_l] + (0.3*P_OW + 0.7)*dat_ad[!, :f_n_pl]))/(1 + Y) + (Ka_PR*dat_ad[!, :AR]))*fup  #lipid

  else  #neutrals

    Kp_all = (dat_all[!, :f_ew] + ((1 + X)/(1 + Y))*dat_all[!, :f_iw] + ((P*dat_all[!, :f_n_l] + (0.3*P + 0.7)*dat_all[!, :f_n_pl]))/(1 + Y) + (Ka_PR*dat_all[!, :LR]))*fup  #non lipid

    Kp_ad = (dat_ad[!, :f_ew] + ((1 + X)/(1 + Y))*dat_ad[!, :f_iw] + ((P_OW*dat_ad[!, :f_n_l] + (0.3*P_OW + 0.7)*dat_ad[!, :f_n_pl]))/(1 + Y) + (Ka_PR*dat_ad[!, :LR]))*fup  #lipid

  end


  Kp = [Kp_ad; Kp_all]

  Kp_df = DataFrame.(Kp_values = Kp)

  Kp_tissue1 = transform(dat_all, :tissue => ByRow(x-> lowercase(x)) => :tissue)

  Kp_tissue2 = "Kp" .* select(Kp_tissue1, :tissue)

  Kp_adipose = DataFrame.(tissue = ["Kpadipose"])


  Kp_tissue = outerjoin(Kp_adipose, Kp_tissue2, on = :tissue)


  Kp_values = hcat(Kp_tissue, Kp_df)


  return(Kp_values)
```

```
end



## Poulin and Theil
human_PT = CSV.File("/home/jrun/data/code/UF_LabLesvi/Data_IN/Kp_Methods/human_PT_Edited.csv",
        missingstring=["", "NA", "."])
human_PT_df = DataFrame(human_PT)

function calcKp_PT(logP, pKa, fup, type, dat)

 dat_all  = dat[findall(in(["Bone","Gut","Heart","Kidney","Liver","Lung","Muscle","Spleen","Skin"]), dat.tissue), :]  #df for all except
Adipose and Plasma
 dat_ad   = filter(row -> row.tissue == ("Adipose"), dat)  #df for Adipose
 dat_plas = filter(row -> row.tissue == ("Plasma"), dat)   #df for Plasma

 Vwp  = dat_plas[1,:f_water]
 Vnlp = dat_plas[1,:f_n_l]
 Vphp = dat_plas[1,:f_pl]

 Vwt   = dat_all[!, :f_water]
 Vwad  = dat_ad[1,:f_water]
 Vnlt  = dat_all[!,:f_n_l]
 Vnlad = dat_ad[1,:f_n_l]
 Vpht  = dat_all[!,:f_pl]
 Vphad = dat_ad[1,:f_pl]
```

```
pH = dat_ad[1,:pH]

logD = 1.115*logP-1.35 #logD is the olive oil:buffer(water) partition coefficient of nonionized species

#Calculate logD_star value
if type==1         #Neutral
  logD_star = logD
elseif type==2     #Monoprotic Acid
  logD_star = logD-log10(1+10^(pH-pKa))
elseif type==3     #Monoprotic Base
  logD_star = logD-log10(1+10^(pKa-pH))
elseif type==4     #Diprotic Acid
  logD_star = logD-log10(1+10^(2*pH-pKa[1]-pKa[2]))
elseif type==5     #Diprotic Base
  logD_star = logD-log10(1+10^(pKa[1]+pKa[2]-2*pH))
elseif type==6     #Monoprotic acid and Monoprotic base (acid comes first)
  logD_star = logD-log10(1+10^(pKa[2]-pKa[1]))
elseif type==7     #Triprotic Acid
  logD_star = logD-log10(1+10^(3*pH-pKa[1]-pKa[2]-pKa[3]))
elseif type==8     #Triprotic Base
  logD_star = logD-log10(1+10^(pKa[1]+pKa[2]+pKa[3]-3*pH))
elseif type==9     #Diprotic acid and Monoprotic base (first two are acid)
  logD_star = logD-log10(1+10^(pH-pKa[1]-pKa[2]+pKa[3]))
elseif type==10    #Monoprotic acid and Diprotic base (first one is acid)
  logD_star = logD-log10(1+10^(pKa[2]+pKa[3]-pKa[1]-pH))
else
```

```
    logD_star = "Type of Compound Not specified Correctly"
  end

  D_star = 10^logD_star
  Kpad   = ((D_star*(Vnlad+0.3*Vphad)+(1*(Vwad+0.7*Vphad)))/(D_star*(Vnlp+0.3*Vphp)+(1*(Vwp+0.7*Vphp)))) * fup

  P = 10^logP
  fut = 1/(1+((1-fup)/fup)*0.5)

  Kpt = ((P*(Vnlt+0.3*Vpht)+(1*(Vwt+0.7*Vpht)))/(P*(Vnlp+0.3*Vphp)+(1*(Vwp+0.7*Vphp)))) * (fup/fut)

  Kp         = [Kpad; Kpt]
  Kp_df      = DataFrame.(Kp_values = Kp)
  Kp_tissue1 = transform(dat_all, :tissue => ByRow(x-> lowercase(x)) => :tissue)
  Kp_tissue2 = "Kp" .* select(Kp_tissue1, :tissue)
  Kp_adipose = DataFrame.(tissue = ["Kpadipose"])

  Kp_tissue  = outerjoin(Kp_adipose, Kp_tissue2, on = :tissue)

  Kp_values  = hcat(Kp_tissue, Kp_df)
  push!(Kp_values,["Kppancreas", 0])

  return(Kp_values)
 end

##Berezhkovisky
```

```
human_Berez = CSV.File("/home/jrun/data/code/UF_LabLesvi/Data_IN/Kp_Methods/human_Berez_Edited.csv",
                missingstring=["", "NA", "."])
human_Berez_df = DataFrame(human_Berez)

function calcKp_Berez(logP, pKa, fup, type, dat)

  dat_all  = dat[findall(in(["Bone","Gut","Heart","Kidney","Liver","Lung","Muscle","Spleen","Skin"]), dat.tissue), :]  #df for all except
Adipose and Plasma
  dat_ad   = filter(row -> row.tissue == ("Adipose"), dat)  #df for Adipose
  dat_plas = filter(row -> row.tissue == ("Plasma"), dat)   #df for Plasma

  Vwp  = dat_plas[1,:f_water]
  Vnlp = dat_plas[1,:f_n_l]
  Vphp = dat_plas[1,:f_pl]

  Vwt   = dat_all[!, :f_water]
  Vwad  = dat_ad[1,:f_water]
  Vnlt  = dat_all[!,:f_n_l]
  Vnlad = dat_ad[1,:f_n_l]
  Vpht  = dat_all[!,:f_pl]
  Vphad = dat_ad[1,:f_pl]

  pH = dat_ad[1,:pH]

  logD = 1.115*logP-1.35 #logD is the olive oil:buffer(water) partition coefficient of nonionized species
```

```
#Calculate logD_star value
if type==1        #Neutral
  logD_star = logD
elseif type==2     #Monoprotic Acid
  logD_star = logD-log10(1+10^(pH-pKa))
elseif type==3     #Monoprotic Base
  logD_star = logD-log10(1+10^(pKa-pH))
elseif type==4     #Diprotic Acid
  logD_star = logD-log10(1+10^(2*pH-pKa[1]-pKa[2]))
elseif type==5     #Diprotic Base
  logD_star = logD-log10(1+10^(pKa[1]+pKa[2]-2*pH))
elseif type==6     #Monoprotic acid and Monoprotic base (acid comes first)
  logD_star = logD-log10(1+10^(pKa[2]-pKa[1]))
elseif type==7     #Triprotic Acid
  logD_star = logD-log10(1+10^(3*pH-pKa[1]-pKa[2]-pKa[3]))
elseif type==8     #Triprotic Base
  logD_star = logD-log10(1+10^(pKa[1]+pKa[2]+pKa[3]-3*pH))
elseif type==9     #Diprotic acid and Monoprotic base (first two are acid)
  logD_star = logD-log10(1+10^(pH-pKa[1]-pKa[2]+pKa[3]))
elseif type==10    #Monoprotic acid and Diprotic base (first one is acid)
  logD_star = logD-log10(1+10^(pKa[2]+pKa[3]-pKa[1]-pH))
else
  logD_star = "Type of Compound Not specified Correctly"
end
```

```
    D_star = 10^logD_star
    Kpad   = ((D_star*(Vnlad+0.3*Vphad)+((Vwad+0.7*Vphad)))/(D_star*(Vnlp+0.3*Vphp)+((Vwp/fup)+0.7*Vphp)))

    P   = 10^logP
    fut = 1/(1+((1-fup)/(2*fup)))

    Kpt = ((P*(Vnlt+0.3*Vpht)+((Vwt/fut)+0.7*Vpht))/(P*(Vnlp+0.3*Vphp)+((Vwp/fup)+0.7*Vphp)))

    Kp         = [Kpad; Kpt]
    Kp_df      = DataFrame.(Kp_values = Kp)
    Kp_tissue1 = transform(dat_all, :tissue => ByRow(x-> lowercase(x)) => :tissue)
    Kp_tissue2 = "Kp" .* select(Kp_tissue1, :tissue)
    Kp_adipose = DataFrame.(tissue = ["Kpadipose"])

    Kp_tissue  = outerjoin(Kp_adipose, Kp_tissue2, on = :tissue)

    Kp_values  = hcat(Kp_tissue, Kp_df)
    push!(Kp_values,["Kppancreas", 0])

    return(Kp_values)
  end

R_R   = calcKp_RR(LogP, pKa, Fupl_human, BP_human, Type_of_compound, 1, human_RR_df)
P_T   = calcKp_PT(LogP, pKa, Fupl_human, Type_of_compound, human_PT_df)
Berez = calcKp_Berez(LogP, pKa, Fupl_human, Type_of_compound, human_Berez_df)
```

```
df_Kp_values = outerjoin(R_R, P_T, Berez, on= :tissue, makeunique=true)
rename!(df_Kp_values, [:tissue, :R_R, :P_T, :Berez])

##Select the best KP Method and add the variable in the Final Kp Method

Best_Kp        = ___
convert_to_kpu = 1  #Divide by unbound fraction if you want to calculate unbound Kp (Kpu), else set to 1
Kp_scalar      = 1

Kpadipose_human = (only(Best_Kp[in.(Best_Kp.tissue, Ref(["Kpadipose"])), :Kp_values])  / convert_to_kpu ) * Kp_scalar
Kpbone_human    = (only(Best_Kp[in.(Best_Kp.tissue, Ref(["Kpbone"])), :Kp_values]) / convert_to_kpu ) * Kp_scalar
Kpgut_human     = (only(Best_Kp[in.(Best_Kp.tissue, Ref(["Kpgut"])), :Kp_values])  / convert_to_kpu ) * Kp_scalar
Kpheart_human   = (only(Best_Kp[in.(Best_Kp.tissue, Ref(["Kpheart"])), :Kp_values])  / convert_to_kpu ) * Kp_scalar
Kpkidney_human  = (only(Best_Kp[in.(Best_Kp.tissue, Ref(["Kpkidney"])), :Kp_values])  / convert_to_kpu ) * Kp_scalar
Kpliver_human   = (only(Best_Kp[in.(Best_Kp.tissue, Ref(["Kpliver"])), :Kp_values])  / convert_to_kpu ) * Kp_scalar
Kplung_human    = (only(Best_Kp[in.(Best_Kp.tissue, Ref(["Kplung"])), :Kp_values])  / convert_to_kpu ) * Kp_scalar
Kpmuscle_human  = (only(Best_Kp[in.(Best_Kp.tissue, Ref(["Kpmuscle"])), :Kp_values])  / convert_to_kpu ) * Kp_scalar
Kpskin_human    = (only(Best_Kp[in.(Best_Kp.tissue, Ref(["Kpskin"])), :Kp_values])  / convert_to_kpu ) * Kp_scalar
Kpspleen_human  = (only(Best_Kp[in.(Best_Kp.tissue, Ref(["Kpspleen"])), :Kp_values])  / convert_to_kpu ) * Kp_scalar
Kprest_human    = ((Kpadipose_human +Kplung_human +Kpbone_human +Kpheart_human +Kpkidney_human +Kpmuscle_human
+Kpskin_human +Kpspleen_human +Kpgut_human +Kpliver_human )/10)

##Permeability Parameters of Brain Same for both Males and Females)
Permeability_bbb   = ___                # Permeability (cm/sec) (Source: Optimization of rat data)
BBB_surface_area   = 150000             # Surface area of the BBB (cm^2)
```

```
    PSb_human = Permeability_bbb*BBB_surface_area*3.6    # PS BBB , Permeability surface area product (L/h) ->  Convert from cm^3/sec to L/hr
    PSe_human = 300                                      # PS brainmass-cranial CSF
    PSc_human = 0.1 * PSb_human                          # PS BCSFB , BBB Surface area of CSF is 1/10th of Brain




## PBPK Model Humans ## ------------------------------------------------------------------------------------------


full_pbpk_model_human_v1 = @model begin
    @param begin
      tvCL       ∈ RealDomain(lower=0.1, upper=100.0)
      tvKa       ∈ RealDomain(lower=0.1, upper=20)
      tvfa       ∈ RealDomain(lower=0.1, upper=1)
      tvlag      ∈ RealDomain(lower=1, upper=60)
      Ω          ∈ PDiagDomain(4)
    end

    @random begin
      η          ~ MvNormal(Ω)
    end

    @covariates Qbrain_human Qbulk_human Qssink_human Qcsink_human Qsout_human Qsin_human Qadipose_human Qbone_human Qheart_human Qkidney_human Qmuscle_human Qskin_human Qspleen_human Qgut_human Qha_human Qliver_human Qrest_human Qlung_human Vbb_human Vccsf_human Vscsf_human Vbm_human Vlung_human Vadipose_human Vbone_human Vheart_human Vkidney_human Vmuscle_human Vskin_human Vspleen_human Vgut_human Vliver_human Vbla_human Vblv_human Vrest_human
```

```
@pre begin

 #------------------------------------------------------------------------------------------------------------------------#

 ## Blood Flows (L/hr)
 Qbrain   = Qbrain_human
 Qbulk    = Qbulk_human
 Qssink   = Qssink_human
 Qcsink   = Qcsink_human
 Qsout    = Qsout_human
 Qsin     = Qsin_human

 Qadipose = Qadipose_human     #Adipose Tissue (L/hr)
 Qbone    = Qbone_human         #Bone (L/hr)
 Qheart   = Qheart_human        #Heart (L/hr)
 Qkidney  = Qkidney_human      #Kidney (L/hr)
 Qmuscle  = Qmuscle_human     #Muscle (L/hr)
 Qskin    = Qskin_human         #Skin (L/hr)
 Qspleen  = Qspleen_human      #Spleen (L/hr)
 Qgut     = Qgut_human          #Gut (L/hr)  [Stomach & Oesophagus, Small Intestine, Large Intestine]
 Qha      = Qha_human           #Hepatic Artery (L/hr)
 Qliver   = Qliver_human        #Liver (L/hr)
 Qrest    = Qrest_human         #Pancreas (L/hr)
 Qlung    = Qlung_human        #Lung (L/hr) [100% of cardiac Output]
```

```
#-----------------------------------------------------------------------------------------------------------------------#

## Volumes of organs (L)
Vbb      =  Vbb_human       #Brain blood (L)
Vccsf    =  Vccsf_human     #Cranial CSF (L)
Vscsf    =  Vscsf_human     #Spinal CSF (L)
Vbm      =  Vbm_human       #Brain mass (L)

Vecf     = 0.29             #Extracellular Fluid, Part of Brain Mass (L)

Vlung    = Vlung_human          #Lung (L)
Vadipose = Vadipose_human   #Adipose (L)
Vbone    = Vbone_human        #Bone (L)
Vheart   = Vheart_human       #Heart (L)
Vkidney  = Vkidney_human    #Kidney (L)
Vmuscle  = Vmuscle_human   #Muscle (L)
Vskin    = Vskin_human         #Skin (L)
Vspleen  = Vspleen_human    #Spleen (L)
Vgut     = Vgut_human          #Gut (L)
Vliver   = Vliver_human       #Liver (L)
Vbla     = Vbla_human          #Arterial (L)
Vblv     = Vblv_human          #Venous (L)
Vrest    = Vrest_human        #Rest of the organs (L)

#-----------------------------------------------------------------------------------------------------------------------#
```

```
## Drug Specific Parameters (carried over from the front, no alteration required here)

## Unboud Fraction of drug
BP      = BP_human
Fubm    = Fubm_human                # Fraction unbound in brain mass
Fupl    = Fupl_human                # Fraction unbound in plasma
Fubb    = Fubb_human                # Fraction unbound in blood
Fuccsf  = Fuccsf_human              # Fraction unbound in cranial CSF
Fuscsf  = Fuscsf_human              # Fraction unbound in spinal CSF

PSb = PSb_human                     #  PS BBB (L/hr) (Converted from cm^3/sec to L/hr)
PSe = PSe_human                     #  PS brainmass-cranial CSF
PSc = PSc_human                     #  PS BCSFB, BBB Surface area of CSF is 1/10th of Brain

Kpadipose = Kpadipose_human
Kplung    = Kplung_human
Kpbone    = Kpbone_human
Kpheart   = Kpheart_human
Kpkidney  = Kpkidney_human
Kpmuscle  = Kpmuscle_human
Kpskin    = Kpskin_human
Kpspleen  = Kpspleen_human
Kpgut     = Kpgut_human
Kpliver   = Kpliver_human
Kprest    = Kprest_human
```

```
    #--------------------------------------------------------------------------------------------------------------------#

    ##PK Parameters

    ##PK Parameters

    CL = (tvCL * exp(η[1])) / BP
    Ka = tvKa * exp(η[2])

  end

  @dosecontrol begin
    lags   = (Dosegutlumen = tvlag * exp(η[3]),)
    bioav  = (Dosegutlumen = tvfa * exp(η[4]),)
  end

  @dynamics begin

    ##Dose Compartment
    Dosegutlumen' = Dosegutlumen*-Ka

    #### Differential Equations are in terms of Concentrations ####
    Cbb'     = (Qbrain*(Cbla-Cbb) + PSb * (Fubm*Cbm - Fubb * Cbb) + PSc*(Fuccsf * Cccsf - Fubb * Cbb)+ Qssink*Cscsf +
Qcsink*Cccsf)/Vbb
    Cbm'     = (PSb * (Fubb * Cbb - Fubm * Cbm) + PSe*(Fuccsf*Cccsf-Fubm*Cbm) - Qbulk*Cbm)/Vbm
```

```
   Cccsf'   = ((PSe * (Fubm*Cbm -Fuccsf*Cccsf)) + PSc * (Fubb*Cbb - Fuccsf*Cccsf) + Qbulk*Cbm + Qsout*Cscsf - Qsin * Cccsf - Qcsink * Cccsf)/ Vccsf

   Cscsf'   = (Qsin*Cccsf - Qsout * Cscsf - Qssink*Cscsf)/Vscsf


   Cblv'    = (Qadipose*(Cadipose/Kpadipose*BP) + Qbone*(Cbone/Kpbone*BP) + Qheart*(Cheart/Kpheart*BP) + Qkidney*(Ckidney/Kpkidney*BP) + Qmuscle*(Cmuscle/Kpmuscle*BP) + Qskin* (Cskin/Kpskin*BP) + Qliver*(Cliver/Kpliver*BP) + Qbrain*(Cbb) + Qrest*(Crest/Kprest*BP) - Qlung*(Cblv) -(CL*Cblv))/Vblv

   Cbla'    = (Qlung*(Clung/Kplung*BP) - Qrest*Cbla - Qbrain*Cbla - Qadipose*Cbla - Qbone*Cbla - Qheart*Cbla - Qkidney*Cbla - Qmuscle*Cbla - Qskin*Cbla - Qspleen*Cbla - Qgut*Cbla - Qha*Cbla)/Vbla

   Clung'   = (Qlung*(Cblv-(Clung/Kplung*BP)))/Vlung

   Cadipose' = (Qadipose*(Cbla-(Cadipose/Kpadipose*BP)))/Vadipose

   Cbone'   = (Qbone*(Cbla-(Cbone/Kpbone*BP)))/Vbone

   Cheart'  = (Qheart*(Cbla-(Cheart/Kpheart*BP)))/Vheart

   Ckidney'  = (Qkidney*(Cbla-(Ckidney/Kpkidney*BP)))/Vkidney

   Cmuscle'  = (Qmuscle*(Cbla-(Cmuscle/Kpmuscle*BP)))/Vmuscle

   Cskin'   = (Qskin*(Cbla-(Cskin/Kpskin*BP)))/Vskin

   Cspleen'  = (Qspleen*(Cbla-(Cspleen/Kpspleen*BP)))/Vspleen

   Crest'   = (Qrest*(Cbla-(Crest/Kprest*BP)))/Vrest

   Cgut'    = (Qgut*(Cbla-(Cgut/Kpgut*BP))+Dosegutlumen*Ka)/Vgut

   Cliver'  = (Qha*Cbla + Qgut*(Cgut/Kpgut*BP) + Qspleen*(Cspleen/Kpspleen*BP) - Qliver*(Cliver/Kpliver*BP))/Vliver

  end


  @derived begin

   "Plasma Concentrations (mg/L)"

   Cpplasma    = @. Cblv/BP

   "ECF Concentrations (mg/L)" # (Assuimg equal distribution in Brain Mass, correcting for volume of extracellular fluid)

   Cecf      = @. Cbm * (Vecf/Vbm)
```

```
    "Spinal CSF Concentrations (mg/L)"
    Cspinal_csf  = @. Cscsf
  end
end

#### PK Parameters for the drug ###

param_v1 =   (tvKa       = ___,
              tvCL       = ___,
              tvfa       = ___,
              tvlag      = ___,
              Ω          = Diagonal([___,___,___,___])
             )

## Dosage Regimen ##

dosage_regimen = DosageRegimen(amt=___, time=___, cmt=___, addl=___, ii=___)
pop           = map(i -> Subject(id=i, events=dosage_regimen, covariates=choose_covariates(i)), 1:Total_Samples)

## Simulation ##
Random.seed!(45668)
sim_v1     = simobs(full_pbpk_model_human_v1, pop, param_v1, obstimes=0:0.1:24)
df         = DataFrame(sim_v1)
dropmissing!(df, :Cpplasma)
df_select  = select(df, :id, :time, :Cpplasma, :Cecf, :Cspinal_csf)
```

```
## calculate the Quantiles
df_quantile = combine(groupby(df_select, :time),
              [n => (x -> (quantile(x, [0.0,0.05,0.50,0.95,1.0]))') => [n*"_q0", n*"_q05",n*"_q50", n*"_q95", n*"_q100"]
              for n in ["Cpplasma", "Cecf", "Cspinal_csf"]])
df_mean = combine(groupby(df_select, :time),
            [col => fun for col in [:Cpplasma, :Cecf, :Cspinal_csf]
            for fun in [mean]])
df_quantile_mean = innerjoin(df_quantile,df_mean, on=[:time])
```
